# Estimating energy expenditure of sperm whales living in social units

**DOI:** 10.1093/conphys/coag024

**Published:** 2026-04-29

**Authors:** Mariana P Silva, Cláudia Oliveira, Rui Prieto, Austin S Allen, Matthew Bowers, Aimee-Kate Darias-O’Hara, Andreas Fahlman, Katarína Klementisová, Madalina Matei, Samantha E Simmons, Mónica A Silva, Leslie New, Sergi Pérez-Jorge

**Affiliations:** Institute of Marine Sciences—OKEANOS & Institute of Marine Research—IMAR, University of the Azores, Rua Professor Doutor Frederico Machado 4, 9901-862 Horta, Portugal; Institute of Marine Sciences—OKEANOS & Institute of Marine Research—IMAR, University of the Azores, Rua Professor Doutor Frederico Machado 4, 9901-862 Horta, Portugal; Institute of Marine Sciences—OKEANOS & Institute of Marine Research—IMAR, University of the Azores, Rua Professor Doutor Frederico Machado 4, 9901-862 Horta, Portugal; Dolphin Quest, 5000 Kahala Avenue, Honolulu, HI 96816,USA; Marine Mammal and Turtle Division, Southeast Fisheries Science Center, National Oceanic and Atmospheric Administration, 75 Virginia Beach Drive, Miami, FL 33149, USA; SMRU Consulting, Scottish Oceans Institute, University of St Andrews, East Sands, St Andrews KY16 8LB, UK; Fundación Oceanogràfic de la Comunidad Valenciana, Valencia 46005, Spain; Global Diving Research, Sanlucar de Barrameda 11540, Spain; IFM, Linkoping University, Linkoping 58153, Sweden; SMRU Consulting, Scottish Oceans Institute, University of St Andrews, East Sands, St Andrews KY16 8LB, UK; SMRU Consulting, Scottish Oceans Institute, University of St Andrews, East Sands, St Andrews KY16 8LB, UK; SMRU Consulting, Scottish Oceans Institute, University of St Andrews, East Sands, St Andrews KY16 8LB, UK; Institute of Marine Sciences—OKEANOS & Institute of Marine Research—IMAR, University of the Azores, Rua Professor Doutor Frederico Machado 4, 9901-862 Horta, Portugal; Department of Mathematics, Computer Science and Statistics, Ursinus College, Collegeville, 601 E Main Street, Collegeville, PA 19426, USA; Institute of Marine Sciences—OKEANOS & Institute of Marine Research—IMAR, University of the Azores, Rua Professor Doutor Frederico Machado 4, 9901-862 Horta, Portugal

**Keywords:** Accelerometry, bioenergetics, energy expenditure, marine mammal, Monte Carlo simulation, physiological ecology, respirometry

## Abstract

Assessing species’ vulnerability to stressors (e.g. changes in prey availability, noise) can be done with bioenergetics models, often within frameworks such as the Population Consequences of Disturbance. However, to successfully quantify the cumulative effects of stressors on individuals it is crucial to understand the link between behavioural change and metabolic costs. Measurements of energy expenditure (e.g. field metabolic rates, FMR) are difficult to obtain for large cetaceans because traditional methods are impractical due to whales’ size. Consequently, energy expenditure must be estimated indirectly, using proxies such as respiration rates and overall dynamic body acceleration (ODBA). Here, we estimated daily FMR of sperm whales (*Physeter macrocephalus*) from social units by combining *in situ* data with these two methods. The estimated mean daily FMR, including estimated basal metabolic rates (BMR), was 412 MJ/day (95% CI: 262.20–616) using the respiration-based method and 620.5 MJ/day (95% CI: 402–839.3) using ODBA. This study provides the first estimates of daily FMR for sperm whales and revealed that averaged-sized individuals from social units have FMRs between 1.59 and 2.39 times the predicted BMR of similar-sized terrestrial mammals, based on respiration rates and ODBA estimates, respectively. Our findings, combined with data on energy acquisition, can contribute to improving predictions of how environmental stressors impact energy balance, health and the long-term population viability of deep-diving marine mammals.

## Abbreviations

FMR: Field metabolic rates
BMR: Basal metabolic rates
ADMR: Average daily metabolic requirement
DLW: Doubly labelled water
*V*_O2_
: Oxygen consumption
*V*_t_
: Tidal volume
*V*_C_
: Tidal capacity
TLC_est_: Estimated total lung capacity
*E*_O2_
: Oxygen exchange fraction
*f*_R_
: Mean respiration rate
*N*_breaths day_
: Estimated number of breaths per day
*M*_body_
: Body mass
*L*_body_
: Body length
ODBA: Overall dynamic body acceleration
Dur_dives_: Dive duration
COL: Cost of locomotion
PCoD: Population Consequences of Disturbance

## Introduction

Animals are constrained by their energetic budgets making energy a relevant proxy for an individual’s overall fitness (Christiansen *et al*., 2014; Grémillet *et al*., 2018). To maximize fitness, animals must balance energy intake and expenditure (Stearns, 1989). Thus, estimates of energy requirements help us understanding life history traits and responses to environmental and human stressors (New *et al*., 2013; Noren and Rosen, 2023). In cetaceans such stressors often trigger behavioural responses, such as reduced foraging, increased locomotion and vocal changes (Steckenreuter *et al*., 2011; Pirotta *et al*., 2015), that ultimately alter energy balance and can impact individual fitness and population viability (Eberhardt, 2002; King *et al*., 2015; Pirotta *et al*., 2018; Farmer *et al*., 2018a). Although these behavioural adjustments affect energy use on short time scales, assessing the cumulative energetic cost over diel cycles provides a more comprehensive measure of the energetic demands of free-ranging animals. Repeated exposure to stressors, especially in species already facing a variety of anthropogenic threats, can create long-term energetic imbalances (Keen *et al*., 2021).

The sperm whale (*Physeter macrocephalus*), globally classified as Vulnerable according to the IUCN Red List (Taylor *et al*., 2019), has not fully recovered from historical whaling (Whitehead and Shin, 2022) and faces multiple anthropogenic stressors, including ship strikes, entanglement, habitat degradation and noise pollution (Gordon *et al*., 1992; Magalhães *et al*., 2002; Richter *et al*., 2003; Azzara *et al*., 2013; Cosentino, 2016; Farmer *et al*., 2018a; Oliveira *et al*., 2022). Among these stressors, some such as acoustic disturbance and vessel traffic have been associated with documented responses including avoidance, changes in dive behaviour and locomotion, reduced foraging, decreased resting and vocal changes (Gordon and Steiner, 1992; Magalhães *et al*., 2002; Richter *et al*., 2003; Cosentino, 2016; Oliveira *et al*., 2022). However, the energetic costs of these responses remain poorly understood, particularly for deep-diving species such as beaked whales (*Ziphiidae*) and sperm whales (Hooker *et al*.,2019; McHuron *et al*., 2022).

Quantifying the energetic costs of sperm whales is essential to predict the energetic responses to stressors (Farmer *et al*., 2018a; Silva *et al*., 2024). Due to their large size and deep-diving behaviour, sperm whales have substantial energy requirements and metabolic demands, potentially increasing their vulnerability to energetic imbalances. Reproductive and social factors (e.g. gestation, lactation, calf attendance, cooperative foraging and maintaining social cohesion) further influence energy allocation. Establishing a baseline of daily energetic costs for social unit individuals provides the foundation to assess how additional reproductive or environmental pressures may alter their energy balance. Understanding these baseline energetic needs, is critical to evaluating the cumulative costs of repeated exposure to stressors.

Field metabolic rates (FMR), the average rate of energy expenditure under field conditions, has been estimated across several marine mammal taxa (Maresh, 2014; Williams *et al*., 2020; Noren and Rosen, 2023). As FMR reflects the total energetic cost it is often contextualized relative to basal metabolic rate (BMR) that represents the minimum energy required to maintain essential physiological functions at rest. Because direct metabolic measurements via respirometry are logistically challenging, particularly in large-bodied species, allometric relationships are often used to estimate BMR. The Kleiber ‘mouse-to-elephant’ curve describes a well-established allometric relationship in which larger animals expend less energy per unit body mass than smaller ones (Kleiber, 1975), and FMR estimates are commonly expressed as multiples of Kleiber-predicted BMR. Across marine mammals, FMR estimates range from 1 to 6 times the Kleiber-predicted BMR. However, there are debates as to whether marine mammals have elevated metabolic rates, given their aquatic adaptation, as these elevated values may reflect thermoregulatory cost, diet or life history traits (Williams *et al*., 2001; Maresh, 2014; Noren and Rosen, 2023). In addition, some studies suggest a smaller allometric scaling exponent relating BMR to body mass in marine mammals, where smaller species expend more energy than expected, while larger ones (>250 kg) expend less than expected (Maresh, 2014; He *et al*., 2023).

FMR estimates vary with behaviour (e.g. dive duration, foraging), context (e.g. lactation) and method of calculation (Arnould *et al*., 1996; Costa and Gales, 2000; Van Der Hoop *et al*., 2014; Fahlman *et al*., 2016; Bejarano *et al*., 2017; McHuron *et al*., 2018; John, 2020; Rimbach *et al*., 2021). In cetaceans, FMR has been measured in small species using doubly labelled water (DLW) and heart rate techniques (Reed *et al*., 2000; Jeanniard-du-Dot *et al*., 2017; Rojano-Donãte *et al*., 2018; Rimbach *et al*., 2021). As these methods are not feasible for large whales, including sperm whales, due to logistical constraints (Butler *et al*., 2004), researchers often extrapolate from scaling equations (Kleiber, 1975; Nagy, 2005), or estimate energy requirements from diet (Bejarano *et al*., 2017; Rojano-Donãte *et al*., 2018). Alternatively, respiration rate and body acceleration can be used as proxies (Halsey *et al*., 2009b; Christiansen *et al*., 2014; Villegas-Amtmann *et al*., 2015; Allen *et al*., 2021).

### Respiration rates

Respiration rates, when combined with physiological respiratory parameters, can estimate FMR based on their correlation with oxygen consumption (V_O2_) (Fick, 1870). The method has been widely applied to cetaceans, particularly medium and large-bodied species, as it relies on breath counts obtained from visual cues or data loggers during surface periods (Christiansen *et al*., 2014; Cosentino, 2016; Roos *et al*., 2016). Although several studies have used fixed values for respiratory parameters such as tidal volume (*V*_t_) and oxygen exchange fraction (*E*_O₂_), (Williams and Noren, 2009; Christiansen *et al*., 2014) these parameters can vary with breathing frequency and activity level (Fahlman *et al*., 2016; Roos *et al*., 2016). Accounting for this variability provides more realistic estimates of metabolic rate under natural conditions.

### Overall dynamic body acceleration

Accelerometry has been used to estimate energetic costs in marine predators (Wilson *et al*., 2020). As movement requires energy, acceleration can reflect the active metabolic cost of locomotion. Metrics such as overall dynamic body acceleration (ODBA) correlate with V_O2_ enabling estimation of movement-related energy expenditure (Wilson *et al*., 2006, 2020; Gleiss *et al*., 2011). Although ODBA does not capture non-locomotion processes such as BMR, non-shivering thermoregulation (Karasov, 1992) or heat increment of feeding (McCue, 2006; Gleiss *et al*., 2011; Wilson *et al*., 2020; Booth *et al*., 2023) with proper calibration ODBA is useful to estimate activity-specific energetic costs (Halsey *et al*., 2009a; Gleiss *et al*., 2011).

### Goal of the study

This study estimates the daily FMR of medium-sized sperm whales by combining *in situ* data with two different methods, respiration rates and ODBA, to address the current lack of empirical data on energy expenditure in large, deep-diving marine mammals. Sperm whales are large cetaceans, with adult females and juveniles typically ranging from 7 to 12 m and males exceeding 14 m (Caruso *et al*., 2015; Giorli and Goetz, 2020; Glarou *et al*., 2023). In the Azores, sperm whales form long-term social units typically composed of related females and their offspring (Christal and Whitehead, 2001; Antunes, 2009; Pinela *et al*., 2009). All individuals tagged in this study were medium-sized and observed in stable groups with calves or other juveniles, consistent with the social structure of females at lower latitudes (Lyrholm *et al*., 1999). We focused on medium-sized individuals from social groups (average length: 8.6 m) because social structure is expected to influence behaviour and energetic requirements. Adult males are largely solitary and inhabit higher latitudes, where environmental conditions and prey fields differ markedly (Whitehead, 2003; Teloni *et al*., 2008; Whitehead, 2018). In contrast, group-living females and juveniles engage in coordinated movements and calf care and may include lactating individuals, which can affect foraging behaviour, dive patterns, recovery time at the surface and overall energy expenditure (Smith and Whitehead, 1993; Gero *et al*., 2013). Focusing on socially structured medium-sized individuals therefore provides demographically consistent FMR estimates and avoids confounding inferences with the distinct behavioural and energetic strategies of solitary adult males.

Filling this knowledge gap provides critical information for bioenergetic models more broadly, improving our understanding of the species’ ecology and energetic requirements. Such estimates are particularly valuable for frameworks like the Population-level Consequences of Disturbance (PCoD), which has been used to link individual-level behavioural and physiological changes to potential population-level impacts and support assessments of aggregate or cumulative effects of anthropogenic stressors on sperm whale populations (e.g. New *et al*., 2013; Pirotta *et al*., 2018; Allen *et al*., 2021; Pirotta, 2022; Fahlman *et al*., 2023).

This study used two different methods to estimate FMR: (i) breathing frequency and (ii) activity. We refined the respiration-based method to allow *V*_t_ and *E*_O₂_ to vary with breathing frequency across surfacing states, which helps improve the existing approach by incorporating uncertainty into the estimates of *V*_t_ and *E*_O2_ (Videsen *et al*., 2023). For estimating FMR from activity, we used acceleration data from the three dive phases (descent, bottom, ascent) and estimated locomotion costs from ODBA during foraging (i.e. diving) and while at the surface. These locomotion cost estimates were combined with estimated BMR to obtain daily FMR. Finally, the FMR estimates from the two methods were compared to the Kleiber-predicted BMR using the mean body mass of medium-sized sperm whales, allowing a better understanding and quantification of the magnitude and variation of FMR across marine mammal groups.

## Materials and Methods

### Tag data

We used audio and movement data collected from seven sperm whales instrumented with DTAGs (Digital Acoustic Recording Tag) (Johnson and Tyack, 2003), deployed using a cantilevered or hand pole (for more details on the tagging procedures see Oliveira *et al*., 2022) (Supplementary Materials: Table S1), during boat-based surveys off the Azores archipelago between 2018 and 2019. Sperm whale tagging was conducted under research permits 37/2016/DRA, 80/2017/DRA and LMAS-DRAM/2018/06 issued by the Regional Government of the Azores. To minimize potential effects from tagging on whale behaviour, the first dive cycle or 1 h of deployment was removed from the dataset (Miller *et al*., 2009). Additionally, the last dive was removed if the tag detached during that dive (four individuals in this study).

### Dive phases and surface periods

**Figure 1:**
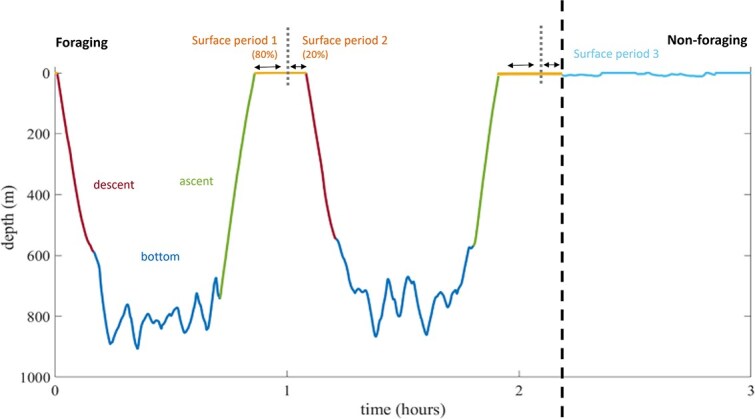
Illustration of a sperm whale dive profile. Dive cycles are composed of descent (dark red), bottom (dark blue), ascent (green) and post-dive surface (orange) phases. Post-dive surface periods between consecutive foraging dives (≤9 min) were divided into two parts: periods 1 (initial 80%) and 2 (final 20%). Surface durations >9 min included a third segment, surface period 3, representing non-foraging time beyond the 9-min threshold, associated with behaviours such as resting, socializing or travelling. Adapted from Oliveira *et al*. (2022).

Foraging dives were identified by the presence of usual clicks and buzzes (Miller *et al*., 2004a; Isojunno and Miller, 2015) and divided into descent, bottom, ascent and post-dive surface phases based on depth profiles and acoustic cues following criteria described in Miller *et al*. (2004b), Watwood *et al*. (2006) and Oliveira *et al*. (2022), using a custom MATLAB script. The post-dive surface phase is considered part of the foraging cycle and associated with recovery between consecutive dives (Fig. 1).

The post-dive surface periods were defined as the interval between consecutive foraging dives (Watwood *et al*., 2006) or, if a dive was followed by a prolonged surface period, the post-dive surface period was standardized to 9 min (orange surface lines in Fig. 1). This threshold was derived from the average post-dive surface durations of 72 dives in the data (Supplementary Materials: Table S1) and aligned with previous reports on sperm whale surfacing behaviour (Papastavrou *et al*., 1989; Gordon and Steiner, 1992; Magalhães *et al*., 2002; Jaquet *et al*., 2000, 2003; Drouot *et al*., 2004; Richter *et al*., 2006; Watwood *et al*., 2006; Gannier *et al*., 2012; Cosentino, 2016). To account for temporal variation in respiratory physiology (*f*_R_, *V*_t_ and *E*_O₂_), post-dive surface periods were divided into surface periods 1 and 2, corresponding to the initial 80% and final 20% of surface time following a dive, respectively (Fig. 1; Supplementary Materials: Fig. S2). A significant difference in *f*_R_ was found between the two periods (Wilcoxon test, *W* = 7353.5, *P* < 0.05) (Table 1), supporting the distinction between surface phases. Surface periods >9 min were composed of three parts: surface periods 1 and 2 (the initial 9 min, maintaining the same physiological distinction as in shorter post-dive periods) and surface period 3. Surface period 3 corresponded to a non-foraging surface period, representing any remaining time beyond the 9-min threshold that was not directly associated with immediate post-dive recovery, but rather with other behaviours such as resting, socializing or travelling (represented in Fig. 1 by the light blue surface line). Sperm whales typically travel at or near the surface during non-foraging surface periods, while movements at depth during deep foraging dives are primarily associated with prey pursuit including bursts of speed during prey capture attempts (Whitehead, 2003; Aoki *et al*., 2012; Fais *et al*., 2016). Although some horizontal displacement can still occur at depth, it is generally interpreted as fine-scale foraging movements in response to prey distribution rather than directed travel (Jaquet *et al*., 2003).

**Table 1:** Parameters used to estimate FMR: mean respiration rate (*f*_R_, measured), tidal volume (*V*_t_, estimated) and oxygen extraction (*E*_O₂_, estimated) for each surfacing period to estimate metabolic rate using a respiration-based method

Surface period	Definition	${\boldsymbol{f}}_{\mathbf{R}}$ (respiration min^−1^)	${\boldsymbol{V}}_{\mathbf{t}}$ (L)	${\mathbf{E}}_{\mathbf{O2}}$ (%)	Daily proportion of time (%)	Daily time (min)
1	Initial 80% of post-dive surfacing time	5.06 (95% CI: 3.78–6.1)	60–80% ${V}_{\mathrm{c}}$	80	9.43 (95% CI: 5.52–13.34)	135.83
2	The remaining 20% of post-dive surfacing time	3.5 (95% CI: 3.2–3.80)	40–50% ${V}_{\mathrm{c}}$	50	2.36 (95% CI:1.38–3.34)	33.96
3	Longer, non-foraging periods at the surface	1.37 (95% CI: 0.81–1.93)	30–40% ${V}_{\mathrm{c}}$	35	29.31 (95% CI: 5.36–53.27)	422.09

Daily proportion (%) and time (min) indicate the proportion and absolute time spent in each surface period per day, calculated from *in situ* tagging data.

### Respiration rates

#### Detection and estimates of respiration rates

Respirations were identified visually and aurally in tag sound recordings. However, recordings from several of the tags used in this study were affected by splash noise, compromising the ability to detect respirations. Therefore, for this component of the analysis, we used data from additional DTAG deployments on individuals that were not part of the seven whales included in the main analysis. These tags were selected because their placement near the blowhole (*n* = 3, Supplementary Materials: Table S3 and Fig. S2) allowed for more accurate detection of the respirations, and recorded for durations ranging between 3.6 and 19 h, covering both daytime and night-time periods. Sperm whales are known to forage throughout the diel cycle, with no consistent differences in diving behaviour or foraging effort between day and night (Watwood *et al*., 2006; Aoki *et al*., 2012). For surface periods 1, 2 and 3 (defined in the previous section as different phases of post-dive recovery and non-foraging surface time), a mean respiration rate (*f*_R_) of 5.06 (95% CI: 3.78–6.1), 3.45 (95% CI: 3.2–3.80) and 1.37 (95% CI: 0.81–1.93) respirations per minute was estimated, respectively (Table 1). These estimates were within the range of sperm whale *f*_R_ values reported from surface observations in the Azores (Gordon and Steiner, 1992; Magalhães *et al*., 2002) and worldwide (Drouot *et al*.,2004; Richter *et al*., 2006; Gannier *et al*., 2012), supporting the representativeness of the data despite the limited sample size.

A lognormal distribution (calculated from DTAGs positioned near the blowhole) was used to model *f*_R_ to constrain the estimate to be positive, and allow for a right skew, allowing for periods of higher respiration rates. The daily number of respirations (*N*_respirations day_) was then calculated by multiplying *f*_R_ by the total amount of time spent in each surface period per day, using the tag recording duration from the seven individuals included in this study (Supplementary Materials: Table S1). This time was calculated by dividing the observed time spent in each surface period on each tag by the tag recording duration and then multiplying it by the number of minutes in a day (Table 1).

#### Rate of oxygen consumption

The volume of oxygen consumed per breath (*V*_O2respiration_, litres O_2_^−1^) was given by the tidal volume (*V*_t_, litres respiration^−1^), and the oxygen extraction (the fraction of oxygen extracted per breath, *E*_O₂_, in %) (Dolphin, 1987; Folkow and Blix, 1992):


(1) \begin{equation*} {V}_{\mathrm{O}2\mathrm{respiration}}={V}_t\times{E}_{O_2} \end{equation*}

These parameters have been measured in small cetaceans housed in human care using breath-by-breath respirometry (Wahrenbrock *et al*., 1974; Fahlman *et al*., 2013). However, it is logistically difficult to perform respirometry in large cetaceans in the wild, requiring assumptions about *V*_t_ and *E*_O₂_ based on body weight and length of animals.

Both *V*_t_ and *E*_O₂_ are related to the animal’s level of exercise and have been demonstrated to vary between and within species (Fahlman *et al*., 2015; Fahlman *et al*., 2016; Roos *et al*., 2016). To deal with the uncertainty in the parameter estimates and with variability in the volume of air inspired during respirations, Videsen *et al*. (2023) estimated a probability distribution for *V*_O2breath_ by modelling the mean and variance of *V*_t_ and *E*_O₂_. The same approach was implemented in this study, using Monte Carlo simulations to estimate the energy expended per respiration.

##### Tidal volume

Tidal volume represents the volume of air inspired per respiration. *V*_t_ has traditionally been assumed equal or close to vital capacity (*V*_C_, in litres) (e.g. 60% of *V*_C_ for grey whales (*Eschrichtius robustus*) in Wahrenbrock *et al*., 1974 and minke whales in Christiansen *et al*. (2014)), which itself is a set proportion of the estimated total lung capacity (TLC_est_, in litres). We assumed that the estimated diving lung volume from the sperm whale (Miller *et al*., 2004b) provided a reliable estimate of the total lung capacity, as:


(2) \begin{equation*} {\mathrm{TLC}}_{\mathrm{est}}=0.026\ {M}_{\mathrm{body}} \end{equation*}

where TLC_est_ was estimated as a function of body mass (M_body_). Equation (2) was derived from data on sperm whales’ body length with measurements ranging from 8.5 to 13.4 m (Miller *et al*., 2004b). In our study, the TLC_est_ was estimated by sampling M_body_ from a distribution of values (Supplementary Materials Table S2, Fig. S1B) as a function of body length, which was estimated from photogrammetry of sperm whales in the Azores (Supplementary Materials Table S2, Fig. S1B, Equation (S1), Lockyer, 1976).

It has been shown that the vital capacity (*V*_c_) of cetaceans is between 80 and 90% of TLC_est_ (Scholander and Irving, 1941; Olsen *et al*., 1969; Fahlman *et al*., 2015; Fahlman *et al*., 2019, thus *V*_C_ was given by the following equation:


(3) \begin{equation*} {V}_c=0.85\ {\mathrm{TLC}}_{\mathrm{est}} \end{equation*}

*V*_t_ was modelled as a proportion of *V*_C_, which varied across each surface period (e.g. periods 1, 2 and 3 as shown in Table 1). We did this to provide a more physiological representation of the temporal response of *V*_t_ following a dive and its correlation with breathing frequency (Van Der Hoop *et al*., 2014; Fahlman *et al*., 2016; Fahlman *et al*., 2019).

##### Oxygen extraction efficiency

E_O₂_ is defined as the fraction of inspired oxygen extracted during a respiratory cycle. It has been previously assumed to be a fixed value of 45% (e.g. for minke whales (*Balaenoptera acutorostrata*); Folkow and Blix, 1992; Blix and Folkow, 1995). However, oxygen extraction can vary substantially depending on diving history, ranging from <10% after a few surface respirations to values as high as 80% following long and deep dives (Ridgway *et al*., 1969; Fahlman *et al*., 2019). This variability makes it difficult to estimate *E*_O₂_ in free-ranging individuals.

Videsen *et al*. (2023) proposed estimating *E*_O₂_ using the lung gas equation under assumed steady-state conditions (blood pH at 7.4 and body temperature at 37°C) which produces values typically ranging from 0.24 to 0.45, with a mean <0.35. Although such steady-state assumptions are unlikely to hold for deep-diving species like sperm whales (rarely in steady state between dives) the resulting *E*_O₂_ range is still within the broad physiological limits observed or inferred in marine mammals. To better capture the natural variability and uncertainty in *E*_O₂_ without relying on steady-state assumptions, we modelled *E*_O₂_ as a beta distribution and the parameters of this distribution were allowed to vary based on the simulated surface period (Table 1).

#### Daily field metabolic rate from respiration rates

Daily energy expenditure was estimated using *f*_R_ during surface periods, including post-dive recovery (Isojunno *et al*., 2016) and non-foraging periods (socializing, resting and travelling) (Oliveira *et al*., 2022) (Fig. 1).

Daily field metabolic rate based on respiration (FMR_resp rates_) was calculated as (Dolphin, 1987; Folkow and Blix, 1992):


(4) \begin{equation*} \mathrm{F}{\mathrm{MR}}_{\mathrm{resp}\ \mathrm{rates}}={V}_{\mathrm{O}2\mathrm{respiration}}\ast 20.1\ast{N}_{\mathrm{resp}\mathrm{irations}\ \mathrm{day}} \end{equation*}

where 20.1 is an energy conversion factor, in kJ l^−1^ O_2_ (Kleiber, 1965), *V*_O2respiration_ is the volume of oxygen consumed per respiration (accounting for differences across surface periods) and *N*_respirations day_ is the total number of breaths per day.

##### Sensitivity analysis for FMR estimates

We conducted a sensitivity analysis to explore how the variability associated with *V*_t_ and *E*_O₂_ might affect the estimates of FMR. Each parameter (*V*_t_ or *E*_O₂_) was systematically varied across a biologically relevant range across the three defined surface periods (1, 2 and 3), while the other was held constant at its default model value (Supplementary Materials Table S4), as were the remaining surface periods (Supplementary Materials Table S4). This allowed us to assess the independent effect of each variation. This approach resulted in six sensitivity scenarios covering variations in both *V*_t_ and *E*_O₂_ (Supplementary materials: Table S4). For each scenario, 1000 Monte Carlo simulations were performed to evaluate the impact of parameter changes on FMR estimates.

### Overall dynamic body acceleration

Previous studies have calculated ODBA by summing the absolute values of the tri-axial acceleration data over a specific interval (Wilson *et al*., 2006). Following this approach, low-frequency acceleration was removed by using a high-pass filter with a cut-off of 0.068 Hz, in accordance with recent recommendations of using 0.4 x the dominant fluking frequency and the dominant sperm whale fluking frequency of 0.17 Hz proposed by Martín López *et al*. (2022). In marine mammals larger than ~3 m, body rotations during swimming dominate the measured dynamic acceleration (e.g. high-pass filtered acceleration) (Martín López *et al*., 2022). This underestimates the accelerative motions caused by muscle action (specific acceleration, SA) in the ODBA. Therefore, SA was calculated by removing body rotations (estimated around the lateral magnetometer axis using a 25-degree angular threshold to exclude rotations when the animal’s rotational axis aligned with the Earth’s magnetic field vector) from the high-pass filtered accelerations (dynamic acceleration, DA in Martín López *et al*., 2022). The resulting SAs were summed to calculate ODBA. In this study, ODBA was calculated for the diving period, comprising the descent, bottom and ascent phases (Oliveira *et al*., 2022; Fig. 1). ODBA was not considered for surface periods due to the influence of sea state on ODBA estimation, which can lead to unreliable estimates (Wilson *et al*., 2020). ODBA was calculated over 1-min intervals and values for each individual were divided by its median value and then multiplied by the median ODBA across individuals (Isojunno and Miller, 2015).

**Table 2:** Parameters used in the modelling approach to convert *f_R_* into estimates of energy turnover based on estimated distributions of *V_t_* and *E*_O₂_

**Parameter**	**Units**	**Abbreviation**	**Distribution, value, equation**	**Reference**
Oxygen consumption per breath	L O_2_^−1^	${V}_{\mathrm{O}2\mathrm{respiration}}$	${V}_{\mathrm{O}2\mathrm{respiration}}=0.21\ast{V}_{\mathrm{t}}\ast{E}_{\mathrm{O}2}$	Dolphin (1987); Folkow and Blix (1992)
Relative content of O_2_ in the inspired air	%		$0.21$	
O_2_ extraction	%	${E}_{\mathrm{O}2}$	Beta distribution, parameters vary by surface period (see Table 1)	Dolphin (1987); Ridgway *et al*. (1969); Wahrenbrock *et al*. (1974); Folkow and Blix (1992); Blix and Folkow (1995)
Respiration rate	Respirations/min	${f}_{\mathrm{R}}$	Empirical data from DTAGs	This study
Tidal volume	l	${V}_{\mathrm{t}}$	Mean as proportion of ${V}_{\mathrm{c}}$, sampled from a uniform distribution; SD = 0.1${V}_{\mathrm{c}}$	Wahrenbrock *et al*. (1974); Folkow and Blix (1992); Blix and Folkow (1995); Videsen *et al*., (2023); Olsen *et al*. (1969)
Vital capacity	l	${V}_{\mathrm{c}}$	${V}_c=0.85\ {\mathrm{TLC}}_{\mathrm{est}}$	Kooyman *et al*. (1981); Olsen *et al*. (1969)
Total lung capacity	l	${\mathrm{TLC}}_{\mathrm{est}}$	${\mathrm{TLC}}_{\mathrm{est}}=0.026\ {M}_{\mathrm{body}}$	Estimated diving lung volume (mean 26.4 l 10^−3^ kg; range 21.9–32.6; SD = 1.1) assumed to represent total lung capacity (Miller *et al*., 2004)
Mean body length	m	${L}_{\mathrm{body}}$	Normal distribution, Mean = 8.61; SD = 0.89 estimated from eight sperm whales using photogrammetry	This study
Mean body mass	kg	${M}_{\mathrm{body}}$	${M}_{\mathrm{body}}=1.25\times 0.0196\ {L_{\mathrm{body}}}^{2.74}$	Lockyer (1976)
Probable total number of respirations estimated over a 24-h period	respirations/day	${N}_{\mathrm{respirations}\ \mathrm{day}}$	Log-normal distribution calculated from ${f}_{\mathrm{R}}$ and total time spent in each activity	This study
Field metabolic rate (rate of energy expenditure)	MJ	$\mathrm{F}{\mathrm{MR}}_{\mathrm{resp}\ \mathrm{rates}}$	$\mathrm{MR}={V}_{\mathrm{O}2}\ast 20.1\ast{N}_{\mathrm{respirations}\ \mathrm{day}}$ Energy conversion factor of $20.1$kJ/l O_2_	Dolphin (1987); Folkow and Blix (1992); Scholander and Irving (1941)

Energy expenditure was inferred from ODBA using an activity-energetic calibration that related mean ODBA and the energetic cost of locomotion (in watts/kg) for bottlenose dolphins (*Tursiops truncatus*) (Equation 5) (Allen *et al*., 2022), adjusted for M_body_ and dive duration (Dur_dives_) (Equation (6)).


(5) \begin{equation*} \mathrm{Locomotion}\ \mathrm{cost}=0.09+9.01\ \mathrm{ODBA} \end{equation*}

(6) \begin{equation*} \mathrm{FM}{\mathrm{R}}_{\mathrm{ODBA}}=\frac{COL\times{M}_{\mathrm{body}}\times{Dur}_{\mathrm{dive}}}{1000}+\mathrm{BMR} \end{equation*}

Given the differences in body length and locomotion between bottlenose dolphins and sperm whales, it is likely that some swimming efficiencies are not accounted for when using this approach for larger whales. For example, we did not explicitly include HIF (heat increment of feeding), as empirical estimates are limited for cetaceans (Koliopoulou *et al*., 2025) and the specific time course, magnitude, dietary differences and physiological differences, such as the potential suppression during foraging, as has been reported in pinnipeds, makes it difficult to provide appropriate values (Sparling *et al*., 2007; Svärd *et al*., 2009; Fortune *et al*., 2013; Booth *et al*., 2023). Additionally, although locomotion cost models have been developed for other large cetaceans (e.g. belugas; John, 2020), their direct application to sperm whales is limited by methodological and biomechanical differences. Specifically, existing models are based on species with continuous swimming and moderate dive depths, whereas sperm whales are intermittent, stroke-and-glide swimmers performing extreme deep dives with prolonged gliding phases. These differences influence drag, buoyancy control and energy allocation, making direct model transfer unreliable. Consequently, the ODBA-based calibration for bottlenose dolphins remains the most suitable available tool for estimating locomotion cost in sperm whales, despite being applied beyond its original calibration range. To account for energy-expending processes that are not part of locomotion costs, locomotion costs estimates were combined with estimates of BMR, using Kleiber predictions (Table 2; Kleiber, 1975). Monte Carlo simulations were then used to generate a distribution of daily FMR for sperm whales from social units.

#### Daily field metabolic rates from ODBA: modelling framework

To infer daily FMR from ODBA (FMR_ODBA_) it is crucial to have information about daily activity patterns. As the DTAG data did not cover full diel cycles, extrapolation for a 24-h period may not be representative, particularly if diel behaviour patterns are present. To address this, a modelling framework was built to simulate ‘typical’ daily activity patterns of the tagged sperm whales (Fig. 1).

Sperm whale foraging behaviour consists of deep dives followed by surface intervals (Amano *et al*., 2003; Watwood *et al*., 2006). Their typical dive cycle includes 40- to 50-min foraging dives to depths of 400–1200 m, followed by 9-min post-dive surface intervals (Papastavrou *et al*., 1989; Drouot *et al*., 2004; Watwood *et al*., 2006). Sperm whales can spend 70–80% of their time engaged in foraging including both diving and surface periods (Gordon and Steiner, 1992; Amano *et al*., 2003; Watwood *et al*., 2006; Oliveira, 2014). Based on this, this study assumed that sperm whales spend ~60% of the day diving, 10% in post-dive surfacing periods and 30% in non-foraging activities such as resting, socializing and travelling (Gordon and Steiner, 1992; Jaquet *et al*., 2000; Watwood *et al*., 2006; Gallo-Reynoso *et al*., 2009). Furthermore, it was assumed that a ‘typical’ sperm whale has a mean dive duration of 45–50 min (Gordon and Steiner, 1992; Jaquet *et al*., 2000, 2003; Drouot *et al*., 2004; Watwood *et al*., 2006; Gannier *et al*., 2012).

**Figure 2:**
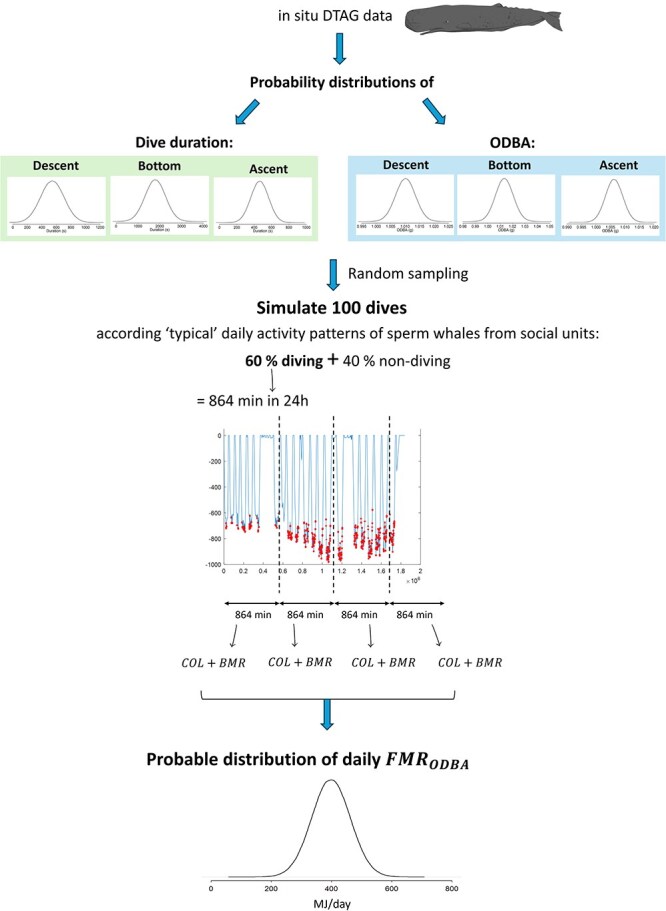
Diagram of modelling framework to estimate daily FMR from ODBA*.* Abbreviations: ODBA, overall dynamic body acceleration; COL, cost of locomotion; BMR, basal metabolic rate; FMR, field metabolic rate.

To represent the variability in diving across individuals, 100 dives were simulated, corresponding to approximately five ‘typical’ days, for 10 individuals (Fig. 2). To account for variability of each dive phase, we drew dive durations from normal distributions and ODBA from log-normal distributions with parameters informed by data on the descent, bottom and ascent phases of dives from the seven tagged sperm whales (Supplementary Materials Fig. S3). Additionally, we incorporated correlations between dive durations across these different dive phases to ensure a more accurate representation of the dive dynamics. A log-normal distribution was used to model ODBA, as it ensures all values remain positive and allows for right-skewed distribution, reflecting the predominance of low-to-moderate effort levels with occasional bursts of high activity. Each individual’s body length was sampled from a normal distribution, whose mean and standard deviation were defined by data available from photogrammetry (Silva *et al*., 2024) of sperm whales sampled in the Azores (Supplementary Materials Fig. S1A and B). Body length was then used to estimate body mass using the mass–length relationship from Lockyer (1976). Each simulated individual was assumed to spend 60% of the time diving (~864 min in 24 h) and a mean FMR_ODBA_ was calculated across all the individuals from their daily simulated data.

For comparative purposes, we also estimated the average daily metabolic requirement (ADMR) following Spitz *et al*. (2018) and Silva *et al*. (2024). ADMR combines BMR (Kleiber, 1947) with a cost-of-living coefficient (β) that accounts for additional energetic costs associated with foraging, thermoregulation and other maintenance and activity costs, excluding reproduction. This coefficient reflects species-specific energetic lifestyles, with β values of 2, 3 or 4 representing low, medium and high energetic cost species, respectively (Spitz *et al*., 2018). For sperm whales, we used β = 2, corresponding to a slow-swimming, low-energy species feeding on low-quality prey (Spitz *et al*., 2012, 2018). ADMR was used as a comparative reference to contextualize FMR estimates derived from respiration rates and ODBA.

All analyses were done using R software version 4.3.2 (R Core Team, 2023). Data processing and manipulation were performed with *dplyr* (Wickham *et al*., 2023), and figures were produced using *ggplot2* (Wickham, 2016). Monte Carlo simulations were implemented using base R functions and custom scripts, including a multivariate lognormal function adapted from the R package *MethylCapSig* (Ayyala *et al*., 2016). As the package is no longer available on CRAN, the function was obtained from the archived online source (rdrr.io/cran/MethylCapSig/src/R/mvlognormal.R; last accessed February 2026). 

## Results

We analysed 84 h of high-resolution biologging data from seven sperm whales instrumented with DTAGs in the Azores. Tag attachment durations varied between 4.46 and 24.67 h (Table S1). All tagged individuals were medium-sized whales encountered with calves or other similar sized whales, thus assumed to belong to social units. To characterize body size, we used photogrammetry-based body length measurements of sperm whales from the same study area as the tagged whales (*n* = 8, unpublished data) (Supplementary Materials: Table S2). Based on these measurements, we created a probability distribution with a mean of 8.61 m (95% CI: 6.86–10.36 m) for the L_body_ for sperm whales in the area (Supplementary Materials Fig. S1A). Then, a probability distribution of M_body_ (mean 9165 kg, 95% CI: 4791–14 846, Supplementary Materials Fig. S1B) was derived from body length values randomly sampled from the distribution of L_body_ (Supplementary Materials: Equation (S1), Lockyer, 1976). Because the relationship between length and mass is non-linear (length raised to the power of 2.74), the resulting mass distribution is skewed, so the 95% confidence interval was calculated directly from the 2.5th and 97.5th percentiles of the simulated mass values rather than assuming a normal distribution.

### Respiration rates

**Figure 3:**
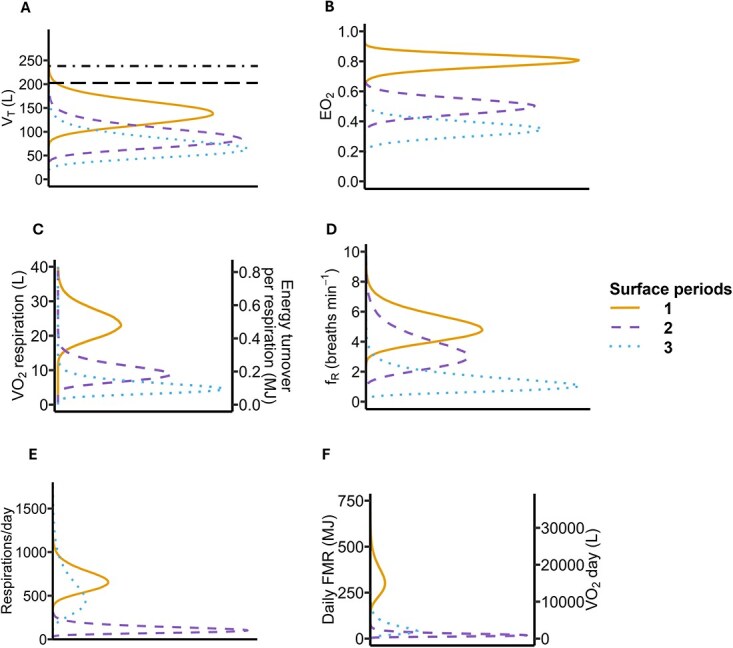
Distributions of parameters used to estimate energy expenditure from respiration rates considering surface periods 1 (solid line), 2 (dashed line) and 3 (dotted line). Parameters were based on measurements and information from previous studies (see Section 1.2 for more details) and assume a mean body mass of ~9163 kg. Each period represents a different type of surface behaviour (see Section 1.3 for details). **(A)** Tidal volume (*V*_t_, in litres). Dashed lines: total lung capacity (TLC_est_, in litres) and vital capacity (VC, in litres). **(B)** Oxygen extraction coefficient (*E*_O_₂, in %). **(C)** The rate of oxygen consumption per breath (*V*_O2 breath_, in litres) and energetic turnover per breath (in MJ). **(D)** Respiration rate (respiration min ^−1^). **(E)** Simulated total respiration count during a day. **(F)** Daily field metabolic rate (FMR_resp rates_) of sperm whales.

**Figure 4:**
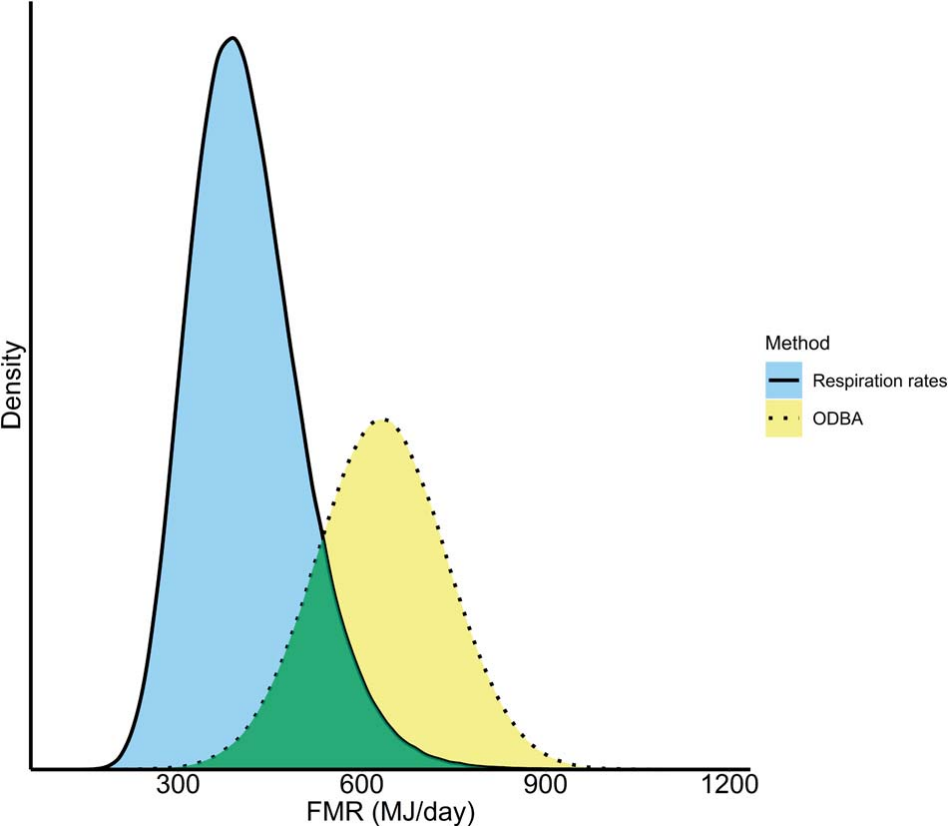
Probability distributions for daily FMR of sperm whales based on 1000 Monte Carlo simulations. The distributions are shown using coloured shading and different line types (blue solid line = FMR_resp rates_ including surface period 1, 2 and 3; yellow dotted line = FMR_ODBA_. The overlapping area is shown in green. The shaded areas represent the full probability distributions and associated uncertainty in the estimates. The area of overlap between the two distributions indicates the range over which the confidence intervals overlap and provides a visual measure of similarity between the two distributions.

The highest respiration rates occurred during surface period 1 (5.06 breaths min^−1^; 95% CI: 3.78–6.1), followed by surface period 2 (3.5 breaths min^−1^; 95% CI: 3.2–3.80) and surface period 3 (1.37 breaths min^−1^; 95% CI: 0.81–1.93) (Fig. 3D). These respiration rates were converted into estimates of energy turnover, using the distributions of *V*_t_ and *E*_O₂_ (Fig. 3C). Mean daily FMR_resp rates_ was estimated at 412.27 MJ (95% CI: 262.20–616) using *f*_R_ from surfacing periods (Table 3, Fig. 4). To scale respiration-based energy turnover to a daily estimate, we used tag-derived proportions of time spent in each surface period to estimate the number of breaths per day. This allowed extrapolation beyond the short recording durations while incorporating variation across individuals and behavioural states. This estimate was 1.59 times the BMR predicted by Kleiber (1975) and 0.79 times the ADMR (Table 3). Surface-specific FMRs varied substantially among periods, with surface period 1-FMR (mean: 328.83 MJ/day; 95% CI: 193.34–517.71) being 14.4 and 5.4 times higher than for surface period 2-FMR (mean: 22.84 MJ/day; 95% CI: 9.44–46.47) and surface period 3-FMR (mean: 60.61 MJ/day; 95% CI: 18.34–148.87), respectively.

**Table 3:** Field metabolic rates estimated for sperm whales from social units

**Parameter**		**Method/context**	**Value (MJ/day)**	**Proportion to BMR Kleiber**	**Proportion to ADMR**	**References**
BMR		$\mathrm{BMR}=293.1\times{M_{\mathrm{body}}}^{0.75}$	272.52 95% CI: 168.98–394.14			Kleiber (1975); Spitz *et al*. (2018))
ADMR		$\mathrm{ADMR}=\mathrm{\beta} \times \mathrm{BMR}$ $\mathrm{\beta} =2\ \mathrm{for}\ \mathrm{sperm}\ \mathrm{whales}$	545.03 95% CI: 337.95–788.27			Spitz *et al*. (2018); Silva *et al*. (2024))
FMR	Respiration rates	Surface period 1	328.83 95% CI: 193.34–517.71			This study
		Surface period 2	22.84 95% CI: 9.44–46.47			
		Total surface following a dive = periods 1 + 2	351.67 95% CI: 214.64–541.52	1.35	0.68	
		Surface period 3	60.61 95% CI: 18.34–148.87	0.23	0.12	
		Total daily: periods 1 + 2 + 3	412.27 95% CI: 262.20–616	1.59	0.79	
	ODBA	From diving *in situ* data	701.1 95% CI: 557.71–844.44	2.70	1.35	
		COL	359.9 95% CI: 211.7–508	1.38	0.7	
		COL + BMR	620.5 95% CI: 402–839.3	2.39	1.19	

Abbreviations: BMR, basal metabolic rate; ADMR, average daily metabolic rate; FMR, field metabolic rate; ODBA, overall dynamic body acceleration; COL, cost of locomotion; M_body_, body mass.

BMR using allometric equation from Kleiber (1975). ADMR (including a species-specific parameter β that accounts for activity costs, assumed to be 2 for sperm whales) (Spitz *et al*., 2018; Silva *et al*., 2024). FMR estimated from respiration rates and ODBA.

FMR estimates varied across the six sensitivity scenarios, with mean values ranging from 365.40 to 409.85 MJ/day (Supplementary Materials: Table S4). Standard deviations remained low across all scenarios while confidence intervals were wider in cases where *V*_t_ covered a broader range. The highest mean FMR estimate (409.85 MJ/day) was observed when *V*_t_ varied between 20 and 60% *V*_c_ and *E*_O₂_ was fixed at 50%. In contrast, the lowest mean estimate (365.40 MJ/day) resulted from a scenario where *V*_t_ ranged from 40 to 80% *V*_c_, with *E*_O₂_ fixed at 80%.

### ODBA

A mean daily FMR_ODBA_ of 620.5 MJ/day (95% CI: 402–839.3) was estimated based on locomotion-related energy expenditure (Table 3, Fig. 4) through a simulation-based approach. The predicted COL accounted for ~58% of this total, corresponding to 359.9 MJ/day (95% CI: 211.7–508) (Table 3). The remaining 42% was allocated to BMR that represents the baseline energy required for maintaining essential physiological functions. FMR_ODBA_ was 2.39 multiples of Kleiber (1975), and 1.19 times the ADMR (Spitz *et al*., 2018; Silva *et al*., 2024) (Table 3).

## Discussion

While FMR has been measured in small- and medium-sized cetaceans under human care (e.g. harbour porpoises, *Phocoena phocoena,*  Rojano-Donãte *et al*., 2018; bottlenose dolphins, Rimbach *et al*., 2021), obtaining estimates for large deep-divers is challenging due to their prolonged submergence time, large size and offshore distribution (Noren and Rosen, 2023). This study provides the first estimates of daily FMR for medium-sized sperm whales in social units, using two methodologies applied to data collected from animal-borne tags (DTAGs). These findings advance the understanding of the energy expenditure of sperm whales within this size class and social structure, contributing to a better understanding of their bioenergetics and providing valuable information for models and frameworks evaluating population-level responses to environmental and anthropogenic pressures (e.g. PCoD, Pirotta *et al*., 2018; Pirotta, 2022). It should be noted that our analysis is based on a relatively small sample size, and only two of the seven tagged individuals provided complete 24-h records. Although the shorter records might not capture the complete range of individual variability, they encompassed representative behavioural states typical of social units in the Azores (Watwood *et al*., 2006; Oliveira, 2014). Thus, while some uncertainty remains, the results likely provide a first approximation of the energetic costs of medium-sized sperm whales, with future studies including longer deployments expected to refine these estimates.

Respiration and acceleration data produced different daily FMR estimates: 412.27 MJ/day (95% CI: 262.20–616) from respiration rates and 620 MJ/day (95% CI: 402–839.3) from ODBA. These two approaches quantify energy expenditure during different behavioural states and therefore capture complementary components of daily metabolic demand. The ODBA-based method estimates locomotion-related energetic costs during diving and foraging, the most energetically demanding phase of the daily cycle and extrapolates these costs to a 24-h period using observed diving time budgets. In contrast, the respiration-based method estimates energy expenditure during surface periods that include post-dive recovery, dive preparation and non-foraging behaviours and integrates total metabolic processing reflected in oxygen consumption. In both cases, daily FMR is derived by combining state-specific metabolic rates with realistic allocations of time across behavioural states. The means of both methods differed by ~33.5%, and although the distributions partially overlap (Fig. 4), such differences may have important implications for applications such as PCoD models, potentially influencing predictions of prey consumption and individual risk; i.e. the probability that an individual’s energetic balance becomes compromised with potential consequences for health, reproduction or survival. Such discrepancies are common and reflect method-specific assumptions and individual variability (He *et al*., 2023; Noren and Rosen, 2023). For example, while ODBA captures movement-related costs it may overlook other metabolic processes, whereas respiration-based estimates integrate total metabolic demand but depend on assumptions about oxygen intake. Although we did not explicitly include HIF in the FMR_ODBA_, recent estimates in bottlenose dolphins suggest HIF is ~8% of BMR. Applying this to sperm whales would add roughly 21.8 MJ/day to the FMR_ODBA_, increasing it by only ~3.5%, from 620.5 MJ/day to ~642.3 MJ/day. This small increase is well within the confidence intervals of our model and does not qualitatively change our conclusions. Nevertheless, future improvements should consider HIF estimates and include validation of ODBA-derived estimates against respirometry and account for individual variability and prey composition as differences in prey quality and energy content affect overall metabolic demand. Recognizing these methodological differences is important when interpreting energetic budgets and improving the accuracy of models used in conservation and management contexts. Similar discrepancies among methods have been observed in other species, even when using ‘gold standard’ methodologies (e.g. DLW, food intake scaling, open flow and breath-by-breath respirometry), including in bottlenose dolphins housed in zoological facilities (Van Der Hoop *et al*., 2014; Fahlman *et al*., 2016; Bejarano *et al*., 2017; John, 2020; Rimbach *et al*., 2021).

Social structure can also influence energy expenditure in sperm whales, and our estimates specifically consider the energetic profiles of females and immatures living in social units. Group-living in these whales is associated with coordinated movement, synchronized diving and resting patterns and cooperative care behaviours (Arnbom and Whitehead, 1989; Whitehead, 1996; Gero *et al*., 2013). Such cohesion may promote energetic efficiency by reducing locomotor costs and optimizing recovery intervals at the surface (Smith and Whitehead, 1993). Conversely, solitary adult males typically forage independently at higher latitudes, perform longer and deeper dives and face greater thermoregulatory challenges (Whitehead, 2003; Teloni *et al*., 2008; Whitehead, 2018) that likely increase their energetic requirements. Therefore, the estimates presented here are most representative of social-unit individuals, and caution should be used when extrapolating these values to other demographic or social contexts.

### Respiration rates

One limitation of respiration-based methods is the lack of species-specific physiological data, often requiring extrapolations from other marine mammals (Williams, 1989; Castellini *et al*., 1992; Yazdi *et al*., 1999; Rojano-Donãte *et al*., 2018). In addition, both *V*_t_ and *E*_O₂_ often covary with breathing frequency, and the correlation depends on dive effort and recovery phase (Ridgway *et al*., 1969; Wahrenbrock *et al*., 1974; Fahlman *et al*., 2023), yet are frequently treated as constants (Folkow and Blix, 1992; Blix and Folkow, 1995; Williams and Noren, 2009). For example, breathing frequency is generally higher immediately following diving or during and shortly after exercise (Isojunno *et al*., 2018; Fahlman *et al*., 2019). Thus, at these times *V*_t_ and *E*_O₂_ have also been shown to increase (Fahlman *et al*., 2019). In this study, we allow *V*_t_ and *E*_O₂_ to vary across surface periods, informed by post-dive respiratory dynamics, to capture variation throughout the surface interval, and perform Monte Carlo simulations to incorporate uncertainty (Ridgway *et al*., 1969; Reed *et al*., 2000; Fahlman *et al*., 2016, 2019; Videsen *et al*., 2023). As expected, FMR was higher during surface period 1 indicating post-dive oxygen replenishment (Isojunno *et al*., 2016; Fahlman *et al*., 2019). In contrast, lower rates during surface period 2 likely represent diminishing recovery effort as whales reach a more stable physiological state before the next dive.

Marine mammals, including sperm whales, adjust breathing frequency at the surface to restore oxygen and eliminate carbon dioxide and nitrogen accumulated during dives (Isojunno *et al*., 2016; Fahlman *et al*., 2019). Their ability to rapidly exchange large lung volumes, especially after diving, enables fast recovery and shorter surface intervals (Boutilier *et al*., 2001; Fahlman *et al*., 2008a, 2016, 2019). For example, bottlenose dolphins can restore O_2_ stores within 1.2 min following a 157-s respiration hold (Fahlman *et al*., 2019). In our model, we assumed that a portion of dive-related metabolic costs are repaid during surface period 1, as this phase represents the immediate post-dive recovery interval characterized by elevated respiratory activity. Evidence from other marine mammal species supports this interpretation; e.g. studies on Steller sea lions and pilot whales have shown increased post-dive ventilation consistent with systematic oxygen debt repayment (Fahlman *et al*., 2008b; Isojunno *et al*., 2018). However, whether sperm whales fully recover their dive-related energetic costs within a single period remains uncertain. Beaked whales, e.g. display extended inter-dive intervals following long dives, likely reflecting recovery from anaerobic metabolism (Joyce *et al*., 2017). Thus, while surface period 1 likely contributes substantially to repaying dive-related costs, complete recovery may span over multiple surface intervals or dive cycles.

FMR was lower during surface period 3, which includes longer non-foraging periods involving socializing, resting and travelling (Oliveira *et al*., 2022). However, combining the various behaviours introduces uncertainty, as energetic demands differ across these activities. For instance, socializing and travelling are more energetically demanding than resting (Rendell and Whitehead, 2001; Tyack *et al*., 2006; Goldbogen *et al*., 2019). Future research should refine behavioural time budgets to improve accuracy.

Sensitivity analysis demonstrated *V*_t_ had greater influence on FMR estimates than *E*_O₂_. When either parameter was held constant, changes in the other parameter had smaller effects, suggesting some degree of interdependence. This is consistent with biological and physiological principles where both parameters contribute to oxygen delivery and use, with *V*_t_ determining how much air (thus oxygen) is brought into the system and *E*_O₂_ how efficiently the available oxygen is extracted and used by tissues. These processes work together, thus, a change in one may enhance or reduce the other’s effect. The analysis quantifies model uncertainty from parameter assumptions rather than actual physiological responses. While this does not provide direct evidence of individual whale physiology, it emphasizes the importance of carefully parameterizing *V*_t_ in respiration-based models.

### ODBA

Using accelerometry as a proxy for energy expenditure carries limitations. ODBA captures only acceleration-induced activity and often requires calibration against direct metabolic measurements, usually in controlled settings and with smaller species. Thus, the exact relationship between *V*_O2respiration_ and ODBA in large animals like sperm whales remains uncertain. However, changes in energy expenditure during movement generally scale proportionally with ODBA (Martín López *et al*., 2022; Wilson *et al*., 2020). Thus, ODBA remains a valid proxy for relative energetic costs, especially for underwater behaviours that cannot be visually monitored (Williams *et al*., 2017). Here, we applied a previously established relationship between activity (ODBA) and *V*_O2respiration_ from bottlenose dolphins (Allen *et al*., 2021; Allen *et al*., 2022) to estimate sperm whale’s daily FMR from foraging dives. Differences in body size, morphology and locomotion efficiency between the two species may explain why ODBA-derived FMR values were higher than those based on respiration rates, as the dolphin-derived calibration may overestimate oxygen consumption in larger, less active divers. Despite these limitations, the relationship between ODBA and *V*_O2respiration_ from bottlenose dolphins has proven robust and has been successfully used to evaluate the energetic impact of stressors in free-ranging animals (Fahlman *et al*., 2023).

### FMR variation across cetaceans

**Figure 5:**
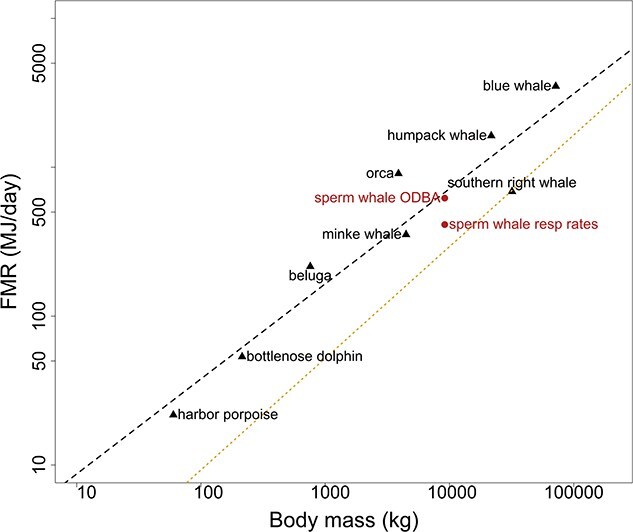
Logarithmic relationship between body mass (*M*_body_) and FMR for sperm whales (circles) and published model-derived estimates for other cetacean species (triangles). Each point represents a species-specific FMR value calculated as the mean when multiple estimates were available. The estimate for the beluga whale represents a theoretical estimate (3x RMR) due to the absence of daily FMR data for this species. Black dashed line is the regression through the cetacean data points. The yellow dotted line is the Kleiber (1975) allometric regression of basal metabolic rate in relation to body mass for terrestrial mammals. Data sources are provided in Tables S5 and S6. This relationship is presented for descriptive purposes and does not incorporate phylogenetic correction thus, comparisons among species should be interpreted cautiously.

Given the challenges of measuring metabolism directly in cetaceans, most available estimates rely on allometric predictions or indirect methods such as DLW, respirometry or modelling approaches. Kleiber's (1975) curve remains a widely used reference to predict BMR across marine mammals, despite the physiological differences from terrestrial species. Although some studies suggest higher BMRs in marine mammals, discrepancies from Kleiber’s predictions vary with species, size and life history (Williams *et al*., 2020;He *et al*., 2023 ; Noren and Rosen, 2023). Metabolic rate scales with body size, where absolute FMR increases but mass-specific FMR decreases, meaning smaller marine mammals expend more energy per kilogramme (He *et al*., 2023; Noren and Rosen, 2023). For example, species <100 kg generally have higher BMRs than terrestrial mammals of the same size, whereas larger species (≥100 kg) exhibit similar BMRs regardless of habitat (He *et al*., 2023). Predicted FMRs follow a similar trend: small species exceed expectations whereas larger species (>250 kg) fall below them (Noren and Rosen, 2023) consistent with our compilation of cetacean data (Fig. 5; Supplementary Materials Fig. S4, Table S5).

Across cetaceans, daily FMR increased with body mass, but the multiples of Kleiber’s FMR tends to decrease in larger species, a pattern indicative of declining mass-specific metabolic demands with increasing size. Smaller odontocetes such as harbour porpoises and bottlenose dolphins showed relatively high FMRs (3–3.5 times Kleiber) consistent with their higher activity levels and thermoregulatory demands (Rojano-Donãte *et al*., 2018; Rimbach *et al*., 2021). The theoretical estimate for beluga (3× RMR (rest metabolic rate), equivalent to ~five times Kleiber) should be interpreted cautiously, as it derives from captive animals and derived from a theoretical framework rather than direct field measurements, and likely reflects different energetic constraints than those of free-ranging individuals (John *et al*., 2024). However, it was included here because to our current knowledge no empirical field-based FMR estimates are available for this species. Larger odontocetes, such as orcas, exhibited FMRs ~6 times Kleiber, reflecting their high activity and locomotion (Noren, 2011). In contrast, our estimates for medium-sized sperm whales (1.59 and 2.39 that of Kleiber, based on respiration rates and ODBA, respectively) place them at the lower end of the range reported for this group, consistent with reduced mass-specific metabolic rates of large-bodied, deep-diving predators (Noren and Rosen, 2023). Mysticetes generally exhibited lower FMR multiples (1–3 times Kleiber) consistent with their large body size and low-cost filter-feeding mode (Videsen *et al*., 2023; Blawas *et al*., 2025). For example, minke, humpback and blue whales had FMRs between 2 and 3× Kleiber (Blix and Folkow, 1995; Blawas *et al*., 2025), whereas southern right whales were approximately equivalent to the Kleiber baseline (~1 time Kleiber) (Christiansen *et al*., 2023). These interspecific differences between odontocetes and mysticetes reflect contrasting locomotor demands, foraging strategies and ecological specializations (Noren and Rosen, 2023).

Methodological heterogeneity among studies further contributes to variability. Empirical techniques (e.g. DLW and respirometry) tend to support conservative estimates and are limited to smaller or captive species, while modelling approaches integrate behavioural and biomechanical data however, depend on assumptions about activity budgets and energy conversion efficiencies. Overall, the compiled cetacean dataset supports the conclusion that FMR scales allometrically with body size, following Kleiber’s (1975) general pattern but with higher interspecific variation driven by ecology, thermoregulation and methodology.

### Applications of the daily FMR estimates

Understanding species-specific energetic needs is essential to accurately parameterize bioenergetic models that inform conservation strategies, especially since environmental and anthropogenic stressors can alter behaviour and physiology, potentially compromising fitness. However, logistical constraints often require indirect methods based on behavioural assumptions or controlled settings that may misrepresent energy use in free-ranging animals (Noren and Rosen, 2023).

Our findings are consistent with physiological and ecological adaptations that allow sperm whales to maintain lower energetic costs than smaller odontocetes. Their large body size facilitates oxygen storage and reduces mass-specific metabolism, while efficient thermoregulation through blubber and a low surface area-to-volume ratio minimizes heat loss (Noren and Williams, 2000; Pabst *et al*., 2016). Their foraging efficiency is reinforced by minimizing transit time during deep dives and using echolocation to detect prey at long distances, adjusting acoustic sampling to prey patchiness (Watwood *et al*., 2006; Fais *et al*., 2015). In contrast, harbour porpoises have higher foraging rates and frequent surfacing, increasing energy demands (Miller *et al*., 2004b; Noren *et al*., 2012; Rojano-Donãte *et al*., 2018; Wisniewska *et al*., 2016). Our FMR estimates for sperm whales support a low cost-of-living strategy, consistent with prior findings on diet quality and muscle composition (Spitz *et al*., 2012). Unlike high-FMR species that require energy-rich species, sperm whales can meet their needs by feeding on low-caloric squid (Spitz *et al*., 2012). Understanding this cost-of-living gradient is crucial for predicting sensitivity to prey fluctuations (Spitz *et al*., 2012; Silva *et al*., 2024).

Our dual-method framework combining respiration and ODBA provides a practical, species-specific approach to estimate daily FMR in deep-diving cetaceans. These outputs when combined with energy acquisition data (Silva *et al*., 2024) can help establish a baseline to assess energetic impacts of stressors. When integrated within the PCoD framework, our study will improve predictions of how environmental and anthropogenic disruptions may affect energy balance and population dynamics in sperm whales.

## Supplementary Material

Web_Material_coag024

## Data Availability

The data and the code underlying this article will be shared on reasonable request to the corresponding author.

## References

[ref1] Allen AS, Read AJ, Shorter KA, Gabaldon J, Blawas AM, Rocho-Levine J, Fahlman A (2022) Dynamic body acceleration as a proxy to predict the cost of locomotion in bottlenose dolphins. J Exp Biol 225(4): jeb243121. 10.1242/jeb.243121. 35014667

[ref2] Allen AS, Read AJ, Wells RS, Allen AS (2021) Estimating the Cost of Locomotion in Common Bottlenose Dolphins: Calibration, Validation, and Application to Study the Impacts of Disturbance. PhD dissertation. Duke University, Durham, NC, USA.

[ref3] Amano M, Yoshioka M, Mori K (2003) Study on diving behavior of sperm whales using suction cup attached TDR tag: an overview. Otsuchi Marine Science 28: 1–5.

[ref4] Antunes R (2009) Variation in Sperm Whale (Physeter macrocephalus) Coda Vocalizations and Social Structure in the North Atlantic Ocean. PhD thesis. University of St Andrews, St Andrews, UK.

[ref5] Aoki K, Amano M, Mori K, Kourogi A, Kubodera T, Miyazaki N (2012) Active hunting by deep-diving sperm whales: 3D dive profiles and maneuvers during bursts of speed. Mar Ecol Prog Ser 444: 289–301. 10.3354/meps09371.

[ref6] Arnbom T, Whitehead H (1989) Observations on the composition and behaviour of groups of female sperm whales near the Galapagos Islands. Can J Zool 67: 1–7. 10.1139/z89-001.

[ref7] Arnould JPY, Boyd IL, Speakman JR (1996) The relationship between foraging behaviour and energy expenditure in Antarctic fur seals. J Zool 239: 769–782. 10.1111/j.1469-7998.1996.tb05477.x.

[ref8] Ayyala DN, Frankhouser DE, Ganbat JO, Marcucci G, Bundschuh R, Yan P, Lin S (2016) Statistical methods for detecting differentially methylated regions based on MethylCap-seq data. Brief Bioinform 17: 926–937. 10.1093/BIB/BBV089. 26454095 PMC5142008

[ref9] Azzara AJ, von Zharen WM, Newcomb JJ (2013) Mixed-methods analytic approach for determining potential impacts of vessel noise on sperm whale click behavior. J Acoust Soc Am 134: 4566–4574. 10.1121/1.4828819. 25669266

[ref10] Bejarano AC, Wells RS, Costa DP (2017) Development of a bioenergetic model for estimating energy requirements and prey biomass consumption of the bottlenose dolphin Tursiops truncatus. Ecol Model 356: 162–172. 10.1016/j.ecolmodel.2017.05.001.

[ref11] Blawas AM, Videsen SKA, Cade DE, Calambokidis J, Friedlaender AS, Johnston DW, Madsen PT, Goldbogen JA (2025) Life in the slowest lane: feeding allometry lowers metabolic rate scaling in the largest whales. Sci Adv 11: eadw2232. 10.1126/sciadv.adw2232. 40768593 PMC12327476

[ref12] Blix AS, Folkow LP (1995) Daily energy expenditure in free living minke whales. Acta Physiol Scand 153: 61–66. 10.1111/j.1748-1716.1995.tb09834.x. 7625169

[ref13] Booth CG, Guilpin M, Darias-O’Hara A-K, Ransijn JM, Ryder M, Rosen D, Pirotta E, Smout S, McHuron EA, Nabe-Nielsen J et al. (2023) Estimating energetic intake for marine mammal bioenergetic models. Conserv Physiol 11: 1–22. 10.1093/conphys/coac083. PMC9900471 36756464

[ref14] Boutilier RG, Reed JZ, Fedak MA (2001) Unsteady-state gas exchange and storage in diving marine mammals: the harbor porpoise and gray seal. Am J Physiol 281: R490–R494. 10.1152/ajpregu.2001.281.2.r490. 11448852

[ref15] Butler PJ, Green JA, Boyd IL, Speakman JR (2004) Measuring metabolic rate in the field: the pros and cons of the doubly labelled water and heart rate methods. Funct Ecol 18: 168–183. 10.1111/j.0269-8463.2004.00821.x.

[ref16] Caruso F, Sciacca V, Bellia G, De Domenico E, Larosa G, Papale E, Pellegrino C, Pulvirenti S, Riccobene G, Simeone F et al. (2015) Size distribution of sperm whales acoustically identified during long term deep-sea monitoring in the Ionian sea. PloS One 10: e0144503. 10.1371/journal.pone.0144503. 26675588 PMC4682957

[ref17] Castellini MA, Kooyman GL, Ponganis PJ (1992) Metabolic rates of freely diving Weddell seals: correlations with oxygen stores, swim velocity and diving duration. J Exp Biol 165: 181–194. 10.1242/jeb.165.1.181. 1588250

[ref18] Christal J, Whitehead H (2001) Social affiliations within sperm whale (Physeter macrocephalus) groups. Ethology 107: 323–340. 10.1046/j.1439-0310.2001.00666.x.

[ref19] Christiansen F, Rasmussen MH, Lusseau D (2014) Inferring energy expenditure from respiration rates in minke whales to measure the effects of whale watching boat interactions. J Exp Mar Biol Ecol 459: 96–104. 10.1016/j.jembe.2014.05.014.

[ref20] Christiansen F, Sprogis KR, Nielsen MLK, Glarou M, Bejder L (2023) Energy expenditure of southern right whales varies with body size, reproductive state and activity level. J Exp Biol 226(13): jeb245137. 10.1242/jeb.245137. 37326244

[ref21] Cosentino AM (2016) Effects of whale-watching vessels on adult male sperm whales off Andenes, Norway. Tour Mar Environ 11: 215–227. 10.3727/154427316X14580612748560.

[ref22] Costa DP, Gales NJ (2000) Foraging energetics and diving behavior of lactating New Zealand sea lions, Phocarctos hookeri. J Exp Biol 203: 3655–3665. 10.1242/jeb.203.23.3655. 11060226

[ref23] Dolphin WF (1987) Dive behavior and estimated energy expenditure of foraging humpback whales in southeast Alaska. Can J Zool 65: 354–362. 10.1139/z87-055.

[ref24] Drouot V, Gannier A, Goold JC (2004) Diving and feeding behaviour of sperm whales (Physeter macrocephalus) in the northwestern Mediterranean Sea. Aquat Mamm 30: 419–426. 10.1578/am.30.3.2004.419.

[ref25] Eberhardt LL (2002) A paradigm for population analysis of long-lived vertebrates. Ecology 83: 2841–2854. 10.1890/0012-9658(2002)083[2841:APFPAO]2.0.CO;2.

[ref26] Fahlman A, Allen AS, Blawas A, Sweeney J, Stone R, Trainor RF, Jensen FH, McHugh K, Allen JB, Barleycorn AA et al. (2023) Surface and diving metabolic rates, and dynamic aerobic dive limits (dADL) in near- and off-shore bottlenose dolphins, Tursiops spp., indicate that deep diving is energetically cheap. Mar Mamm Sci 39: 976–993. 10.1111/mms.13023.

[ref27] Fahlman A, Brodsky M, Miedler S, Dennison S, Ivančić M, Levine G, Rocho-Levine J, Manley M, Rocabert J, Borque-Espinosa A (2019) Ventilation and gas exchange before and after voluntary static surface breath-holds in clinically healthy bottlenose dolphins, Tursiops truncatus. J Exp Biol 222(5): jeb192211. 10.1242/jeb.192211. 30760549

[ref28] Fahlman A, Loring SH, Levine G, Rocho-Levine J, Austin T, Brodsky M (2015) Lung mechanics and pulmonary function testing in cetaceans. J Exp Biol 218: 2030–2038. 10.1242/jeb.119149. 26157159

[ref29] Fahlman A, Van Der Hoop J, Moore MJ, Levine G, Rocho-Levine J, Brodsky M (2016) Estimating energetics in cetaceans from respiratory frequency: why we need to understand physiology. Biol Open 5: 436–442. 10.1242/bio.017251. 26988759 PMC4890674

[ref30] Fahlman A, Svärd C, Rosen DAS, Jones DR, Trites AW (2008a) Metabolic costs of foraging and the management of O2 and CO 2 stores in Steller sea lions. J Exp Biol 211: 3573–3580. 10.1242/jeb.023655. 18978221

[ref31] Fahlman A, Svärd C, Rosen DAS, Wilson RP, Trites AW (2013) Activity as a proxy to estimate metabolic rate and to partition the metabolic cost of diving vs. breathing in pre- and post-fasted Steller sea lions. Aquat Biol 18: 175–184. 10.3354/ab00500.

[ref32] Fahlman A, Wilson R, Svärd C, Rosen DAS, Trites AW (2008b) Activity and diving metabolism correlate in Steller sea lion Eumetopias jubatus. Aquat Biol 2: 75–84. 10.3354/ab00039.

[ref33] Fais A, Aguilar Soto N, Johnson M, Pérez-González C, Miller PJO, Madsen PT (2015) Sperm whale echolocation behaviour reveals a directed, prior-based search strategy informed by prey distribution. Behav Ecol Sociobiol 69: 663–674. 10.1007/s00265-015-1877-1.

[ref34] Fais A, Johnson M, Wilson M, Aguilar Soto N, Madsen PT (2016) Sperm whale predator-prey interactions involve chasing and buzzing, but no acoustic stunning. Sci Rep 6: 1–13. 10.1038/srep28562. 27340122 PMC4919788

[ref35] Farmer NA, Baker K, Zeddies DG, Denes SL, Noren DP, Garrison LP, Machernis A, Fougères EM, Zykov M (2018a) Population consequences of disturbance by offshore oil and gas activity for endangered sperm whales (Physeter macrocephalus). Biol Conserv 227: 189–204. 10.1016/j.biocon.2018.09.006.

[ref36] Farmer NA, Noren DP, Fougères EM, Machernis A, Baker K (2018b) Resilience of the endangered sperm whale Physeter macrocephalus to foraging disturbance in the Gulf of Mexico, USA: a bioenergetic approach. Mar Ecol Prog Ser 589: 241–261. 10.3354/meps12457.

[ref37] Fick A (1870) Ueber die Messung des Blutquantum in den Herzventrikeln. Sb Phys Med Ges Worzburg 16–17.

[ref38] Folkow LP, Blix AS (1992) Metabolic rates of minke whales. Acta Physiol Scand 146: 141–150. 10.1111/j.1748-1716.1992.tb09402.x. 1442122

[ref39] Fortune SME, Trites AW, Mayo CA, Rosen DAS, Hamilton PK (2013) Energetic requirements of North Atlantic right whales and the implications for species recovery. Mar Ecol Prog Ser 478: 253–272. 10.3354/meps10000.

[ref40] Gallo-Reynoso J-P, Égido-Villarreal J, Coria-Galindo E-M (2009) Sperm whale distribution and diving behaviour in relation to presence of jumbo squid in Guaymas Basin, Mexico. Mar Biodivers Rec 2: 1–6. 10.1017/s1755267209990571.

[ref41] Gannier A, Petiau E, Dulau V, Rendell L (2012) Foraging dives of sperm whales in the north-western Mediterranean Sea. J Mar Biol Assoc UK 92: 1799–1808. 10.1017/S0025315412001087.

[ref42] Gero S, Gordon J, Whitehead H (2013) Calves as social hubs: dynamics of the social network within sperm whale units. Proc R Soc B: Biol Sci 280: 20131113. 10.1098/rspb.2013.1113. PMC3774244 23740785

[ref43] Giorli G, Goetz KT (2020) Acoustically estimated size distribution of sperm whales (Physeter macrocephalus) off the east coast of New Zealand. N Z J Mar Freshw Res 54: 177–188. 10.1080/00288330.2019.1679843.

[ref44] Glarou M, Gero S, Frantzis A, Brotons JM, Vivier F, Alexiadou P, Cerdà M, Pirotta E, Christiansen F (2023) Estimating body mass of sperm whales from aerial photographs. Mar Mamm Sci 39: 251–273. 10.1111/mms.12982.

[ref45] Gleiss AC, Wilson RP, Shepard ELC (2011) Making overall dynamic body acceleration work: on the theory of acceleration as a proxy for energy expenditure. Methods Ecol Evol 2: 23–33. 10.1111/j.2041-210X.2010.00057.x.

[ref46] Goldbogen JA, Cade DE, Wisniewska DM, Potvin J, Segre PS, Savoca MS, Hazen EL, Czapanskiy MF, Kahane-Rapport SR, DeRuiter SL et al. (2019) Why whales are big but not bigger: physiological drivers and ecological limits in the age of ocean giants. Science 366: 1367–1372. 10.1126/science.aax9044. 31831666

[ref47] Gordon J, Leaper R, Hartley FG, Chappell O (1992) Effects of whale watching vessels on the surface and underwater acoustic behaviour of sperm whales off Kaikoura, New Zealand. Science and Research Series 52: 1–64.

[ref48] Gordon J, Steiner L (1992) Ventilation and dive patterns in sperm whales, Physeter macrocephalus, in the Azores. Report of the International Whaling Commission 42: 561–565.

[ref49] Grémillet D, Lescroël A, Ballard G, Dugger KM, Massaro M, Porzig EL, Ainley DG (2018) Energetic fitness: field metabolic rates assessed via 3D accelerometry complement conventional fitness metrics. Funct Ecol 32: 1203–1213. 10.1111/1365-2435.13074.

[ref50] Halsey LG, Green JA, Wilson RP, Frappell PB (2009a) Accelerometry to estimate energy expenditure during activity: best practice with data loggers. Physiol and Biochem Zoology 82: 396–404. 10.1086/589815. 19018696

[ref51] Halsey LG, Shepard ELC, Quintana F, Gomez Laich A, Green JA, Wilson RP (2009b) The relationship between oxygen consumption and body acceleration in a range of species. Comp Biochem Physiol A Mol Integr Physiol 152: 197–202. 10.1016/j.cbpa.2008.09.021. 18854225

[ref52] He RS, De Ruiter S, Westover T, Somarelli JA, Blawas AM, Dayanidhi DL, Singh A, Steves B, Driesinga S, Halsey LG et al. (2023) Allometric scaling of metabolic rate and cardiorespiratory variables in aquatic and terrestrial mammals. Physiol Rep 11: e15698. 10.14814/phy2.15698. 37271741 PMC10239733

[ref53] Hooker SK, De Soto NA, Baird RW, Carroll EL, Claridge D, Feyrer L, Miller PJO, Onoufriou A, Schorr G, Siegal E et al. (2019) Future directions in research on beaked whales. Front Mar Sci 5: 514. 10.3389/fmars.2018.00514.

[ref54] Isojunno S, Aoki K, Curé C, Kvadsheim PH, O’Malley Miller PJ (2018) Breathing patterns indicate cost of exercise during diving and response to experimental sound exposures in long-finned pilot whales. Front Physiol 9: 1462. 10.3389/fphys.2018.01462. PMC6232938 30459631

[ref55] Isojunno S, Curé C, Kvadsheim PH, Lam FPA, Tyack PL, Wensveen PJ, Miller PJOM (2016) Sperm whales reduce foraging effort during exposure to 1-2 kH z sonar and killer whale sounds. Ecol Appl 26: 77–93. 10.1890/15-0040. 27039511

[ref56] Isojunno S, Miller PJO (2015) Sperm whale response to tag boat presence: biologically informed hidden state models quantify lost feeding opportunities. Ecosphere 6: 1–46. 10.1890/ES14-00130.1.

[ref57] Jaquet N, Dawson S, Slooten E (2000) Seasonal distribution and diving behaviour of male sperm whales off Kaikoura: foraging implications. Can J Zool 78: 407–419. 10.1139/cjz-78-3-407.

[ref58] Jaquet N, Gendron D, Coakes A (2003) Sperm whales in the Gulf of California: residency, movements, behavior, and the possible influence of variation in food supply. Mar Mamm Sci 19: 545–562. 10.1111/j.1748-7692.2003.tb01320.x.

[ref59] Jeanniard-du-Dot T, Trites AW, Arnould JPY, Speakman JR, Guinet C (2017) Activity-specific metabolic rates for diving, transiting, and resting at sea can be estimated from time–activity budgets in free-ranging marine mammals. Ecol Evol 7: 2969–2976. 10.1002/ece3.2546. 28479996 PMC5415512

[ref60] John JS (2020) Energetics of Rest and Locomotion in Diving Marine Mammals: Novel Metrics for Predicting the Vulnerability of Threatened Cetacean, Pinniped, and Sirenian species. University of California, Santa Cruz

[ref61] John JS, Christen DR, Flammer KL, Kendall TL, Nazario EC, Richter BP, Gill V, Williams TM (2024) Conservation energetics of beluga whales: using resting and swimming metabolism to understand threats to an endangered population. J Exp Biol 227(5): jeb246899. 10.1242/jeb.246899. PMC11070638 38483264

[ref62] Johnson MP, Tyack PL (2003) A digital acoustic recording tag for measuring the response of wild marine mammals to sound. IEEE J Ocean Eng 28: 3–12. 10.1109/JOE.2002.808212.

[ref63] Joyce TW, Durban JW, Claridge DE, Dunn CA, Fearnbach H, Parsons KM, Andrews RD, Ballance LT (2017) Physiological, morphological, and ecological tradeoffs influence vertical habitat use of deep-diving toothed-whales in the Bahamas. PloS One 12: e0185113. 10.1371/journal.pone.0185113. 29020021 PMC5636075

[ref64] Karasov WH (1992) Daily energy expenditure and the cost of activity in mammals. Integr Comp Biol 32: 238–248. 10.1093/icb/32.2.238.

[ref65] Keen KA, Beltran RS, Pirotta E, Costa DP (2021) Emerging themes in population consequences of disturbance models. Proc the R Soc B Biol Sci 288: 20210325. 10.1098/rspb.2021.0325. PMC8385386 34428966

[ref66] King SL, Schick RS, Donovan C, Booth CG, Burgman M, Thomas L, Harwood J (2015) An interim framework for assessing the population consequences of disturbance. Methods Ecol Evol 6: 1150–1158. 10.1111/2041-210X.12411.

[ref67] Kleiber M (1965) Respiratory Exchange and Metabolic Rate. In Handbook of Physiology, Respiration, 3. American Physiological Society, Washington, DC, USA, pp. 927–937.

[ref68] Kleiber M (1975) Metabolic turnover rate: a physiological meaning of the metabolic rate per unit body weight. J Theor Biol 53: 199–204. 10.1016/0022-5193(75)90110-1. 1195755

[ref69] Kleiber M (1947) Body size and metabolic rate. Physiol Rev 27: 511–541. 10.1152/physrev.1947.27.4.511. 20267758

[ref70] Koliopoulou I, DeRuiter SL, Altimiras J, Larsson J, Arenarez J, Rosen D, Fahlman A (2025) Heat increment of feeding in the common bottlenose dolphin (Tursiops truncatus) contributes moderately to field metabolic rate estimates. J Exp Biol 228: jeb251474. 10.1242/jeb.251474. 41185930 PMC12752490

[ref71] Kooyman GL, Diego S, Jolla L, Road SS (1981) Flow properties of expiration and inspiration in a trained bottle-nosed porpoise. Physiol Zool 54: 55–61. 10.1086/physzool.54.1.30155804.

[ref72] Lockyer C (1976) Body weights of some species of large whales. ICES J Mar Sci 36: 259–273. 10.1093/icesjms/36.3.259.

[ref73] Lyrholm T, Leimar O, Johanneson B, Gyllensten U (1999) Sex-biased dispersal in sperm whales: contrasting mitochondrial and nuclear genetic structure of global populations. Proc R Soc B Biol Sci 266: 347–354. 10.1098/rspb.1999.0644. PMC1689695 10097396

[ref74] Magalhães S, Ui Prieto R, Silva A, Oão Gonçalves J, Afonso-Dias M, Santos S, Prieto R, Silva MA, Gonçalves J, Afonso-Dias M et al. (2002) Short-term reactions of sperm whales (Physeter macrocephalus) to whale-watching vessels in the Azores. Aquat Mamm 28: 267–274.

[ref75] Maresh JL (2014). Bioenergetics of Marine Mammals: The Influence of Body Size, Reproductive Status, Locomotion and Phylogeny on Metabolism. University of California, Santa Cruz. https://escholarship.org/uc/item/3c5785r3

[ref76] Martín López LM, Aguilar de Soto N, Madsen PT, Johnson M (2022) Overall dynamic body acceleration measures activity differently on large versus small aquatic animals. Methods Ecol Evol 13: 447–458. 10.1111/2041-210X.13751.

[ref77] McCue MD (2006) Specific dynamic action: a century of investigation. Comp Biochem Physiol A Mol Integr Physiol 144: 381–394. 10.1016/j.cbpa.2006.03.011. 16716621

[ref78] McHuron EA, Adamczak S, Arnould JPY, Ashe E, Booth C, Don Bowen W, Christiansen F, Chudzinska M, Costa DP, Fahlman A et al. (2022) Key questions in marine mammal bioenergetics. Conserv Physiol 10: coac055. 10.1093/conphys/coac055. 35949259 PMC9358695

[ref79] McHuron EA, Peterson SH, Hückstädt LA, Melin SR, Harris JD, Costa DP (2018) The energetic consequences of behavioral variation in a marine carnivore. Ecol Evol 8: 4340–4351. 10.1002/ece3.3983. 29721302 PMC5916299

[ref80] Miller PJO, Johnson MP, Madsen PT, Biassoni N, Quero M, Tyack PL (2009) Using at-sea experiments to study the effects of airguns on the foraging behavior of sperm whales in the Gulf of Mexico. Deep Sea Res Part 1 Oceanogr Res Pap 56: 1168–1181. 10.1016/j.dsr.2009.02.008.

[ref81] Miller PJOO, Johnson MP, Tyack PL (2004a) Sperm whale behaviour indicates the use of echolocation click buzzes “creaks” in prey capture. Proc R Soc B Biol Sci 271: 2239–2247. 10.1098/rspb.2004.2863. PMC1691849 15539349

[ref82] Miller PJOO, Johnson MP, Tyack PL, Terray EA (2004b) Swimming gaits, passive drag and buoyancy of diving sperm whales Physeter macrocephalus. J Exp Biol 207: 1953–1967. 10.1242/jeb.00993. 15107448

[ref83] Nagy KA (2005) Field metabolic rate and body size. J Exp Biol 208: 1621–1625. 10.1242/jeb.01553. 15855393

[ref84] New LF, Moretti DJ, Hooker SK, Costa DP, Simmons SE (2013) Using energetic models to investigate the survival and reproduction of beaked whales (family Ziphiidae). PloS One 8: e68725. 10.1371/journal.pone.0068725. 23874737 PMC3714291

[ref85] Noren DP (2011) Estimated field metabolic rates and prey requirements of resident killer whales. Mar Mamm Sci 27: 60–77. 10.1111/j.1748-7692.2010.00386.x.

[ref86] Noren SR, Kendall T, Cuccurullo V, Williams TM (2012) The dive response redefined: underwater behavior influences cardiac variability in freely diving dolphins. J Exp Biol 215: 2735–2741. 10.1242/jeb.069583. 22837445

[ref87] Noren SR, Rosen DAS (2023) What are the metabolic rates of marine mammals and what factors impact this value: a review. Conserv Physiol 11: 1–19. 10.1093/conphys/coad077. PMC10545007 37790839

[ref88] Noren SR, Williams TM (2000) Body size and skeletal muscle myoglobin of cetaceans: adaptations for maximizing dive duration. Comp Biochem Physiol A Mol Integr Physiol 126: 181–191. 10.1016/S1095-6433(00)00182-3. 10936758

[ref89] Oliveira C, Pérez-Jorge S, Prieto R, Wensveen PJ, Silva MAMA, Cascão I, Wensveen PJ, Silva MAMA (2022) Exposure to whale watching vessels affects dive ascents and resting behavior in sperm whales. Front Mar Sci 9: 1–14. 10.3389/fmars.2022.914397.35450130

[ref90] Oliveira CIB (2014) Behavioural ecology of the sperm whale (*Physeter macrocephalus*) in the North Atlantic Ocean. PhD thesis. Universidade dos Açores, Ponta Delgada, Azores, Portugal.

[ref91] Olsen CR, Hale FC, Elsner R (1969) Mechanics of ventilation in the pilot whale. Respir Physiol 7: 137–149. 10.1016/0034-5687(69)90001-2. 5823828

[ref92] Pabst DA, McLellan WA, Rommel SA (2016) How to build a deep diver: the extreme morphology of mesoplodonts. Integr Comp Biol 56: 1337–1348. 10.1093/icb/icw126. 27940620

[ref93] Papastavrou V, Smith SC, Whitehead H (1989) Diving behaviour of the sperm whale, Physeter macrocephalus, off the Galapagos Islands. Can J Zool 67: 839–846. 10.1139/z89-124.

[ref94] Pinela AM, Quérouil S, Magalhães S, Silva MA, Prieto R, Matos JA, Santos RS (2009) Population genetics and social organization of the sperm whale (Physeter macrocephalus) in the Azores inferred by microsatellite analyses. Can J Zool 87: 802–813. 10.1139/Z09-066.

[ref95] Pirotta E (2022) A review of bioenergetic modelling for marine mammal populations. Conserv Physiol 10: 1–16. 10.1093/conphys/coac036. PMC9215292 35754757

[ref96] Pirotta E, Booth CG, Costa DP, Fleishman E, Kraus SD, Lusseau D, Moretti D, New LF, Schick RS, Schwarz LK et al. (2018) Understanding the population consequences of disturbance. Ecol Evol 8: 9934–9946. 10.1002/ece3.4458. 30386587 PMC6202709

[ref97] Pirotta E, Thompson PM, Cheney B, Donovan CR, Lusseau D (2015) Estimating spatial, temporal and individual variability in dolphin cumulative exposure to boat traffic using spatially explicit capture-recapture methods. Anim Conserv 18: 20–31. 10.1111/acv.12132.

[ref98] Reed JZ, Chambers C, Hunter CJ, Lockyer C, Kastelein R, Fedak MA, Boutilier RG (2000) Gas exchange and heart rate in the harbour porpoise, Phocoena phocoena. J Comp Physiol B Biochem Syst Environ Physiol 170: 1–10. 10.1007/s003600050001. 10707319

[ref99] Rendell L, Whitehead H (2001) Culture in whales and dolphins. Behav Brain Sci 24: 309–382. 10.1016/B978-0-12-373553-9.00068-7. 11530544

[ref100] Richter C, Dawson S, Slooten E (2006) Impacts of commercial whale watching on male sperm whales at Kaikoura. New Zealand Marine Mammal Science 22: 46–63. 10.1111/j.1748-7692.2006.00005.x.

[ref101] Richter CF, Dawson SM, Slooten E (2003) Sperm whale watching off Kaikoura, New Zealand: effects of current activities on surfacing and vocalisation patterns. Scir Conserv 219: 5–78.

[ref102] Ridgway SH, Scronce BL, Kanwisher J (1969) Respiration and deep diving in the bottlenose porpoise. Science 166: 1651–1654. 10.1126/science.166.3913.1651. 5360592

[ref103] Rimbach R, Amireh A, Allen A, Hare B, Guarino E, Kaufman C, Salomons H, Pontzer H (2021) Total energy expenditure of bottlenose dolphins (*Tursiops truncatus*) of different ages. J Exp Biol 224(15): jeb242218. 10.1242/jeb.242218. 34350948

[ref104] Rojano-Donãte L, McDonald BI, Wisniewska DM, Johnson M, Teilmann J, Wahlberg M, Højer-Kristensen J, Madsen PT (2018) High field metabolic rates of wild harbour porpoises. J Exp Biol 221(23): jeb189688. 10.1242/jeb.185827. 30523043

[ref105] Roos MMH, Wu GM, Miller PJO (2016) The significance of respiration timing in the energetics estimates of free-ranging killer whales (Orcinus orca). J Exp Biol 219: 2066–2077. 10.1242/jeb.137513. 27385756

[ref106] Scholander PF, Irving L (1941) Experimental investigations on the respiration and diving of the Florida manatee. J Cell Comp Physiol 17: 169–191. 10.1002/jcp.1030170204.

[ref107] Silva MP, Oliveira C, Prieto R, Silva MA, New L, Pérez-Jorge S (2024) Bioenergetic modelling of a marine top predator’s responses to changes in prey structure. Ecol Evol 14: e11135. 10.1002/ece3.11135. 38529024 PMC10961477

[ref108] Smith SC, Whitehead H (1993) Variations in the feeding success and behaviour of Galapagos sperm whales (Physeter macrocephalus) as they relate to oceanographic conditions. Can J Zool 71: 1991–1996. 10.1139/z93-283.

[ref109] Sparling CE, Fedak MA, Thompson D (2007) Eat now, pay later? Evidence of deferred food-processing costs in diving seals. Biol Lett 3: 95–99. 10.1098/rsbl.2006.0566. PMC2373816 17443975

[ref110] Spitz J, Ridoux V, Trites AW, Laran S, Authier M (2018) Prey consumption by cetaceans reveals the importance of energy-rich food webs in the Bay of Biscay. Prog Oceanogr 166: 148–158. 10.1016/j.pocean.2017.09.013.

[ref111] Spitz J, Trites AW, Becquet V, Brind’Amour A, Cherel Y, Galois R, Ridoux V (2012) Cost of living dictates what whales, dolphins and porpoises eat: the importance of prey quality on predator foraging strategies. PloS One 7: e50096. 10.1371/journal.pone.0050096. 23185542 PMC3503768

[ref112] Stearns SC (1989) Trade-offs in life-history evolution. Funct Ecol 3: 259–268. 10.2307/2389364.

[ref113] Steckenreuter A, Möller L, Harcourt R (2011) How does Australia’ s largest dolphin-watching industry affect the behaviour of a small and resident population of Indo-Pacific bottlenose dolphins? J Environ Manage 97: 14–21. 10.1016/j.jenvman.2011.11.002. 22325578

[ref114] Svärd C, Fahlman A, Rosen DAS, Joy R, Trites AW (2009) Fasting affects the surface and diving metabolic rates of Steller sea lions Eumetopias jubatus. Aquat Biol 8: 71–82. 10.3354/ab00211.

[ref115] Taylor BL, Baird R, Barlow J, Dawson SM, Ford J, Mead JG, Notarbartolo di Sciara G, Wade P, Pitman R (2019). Physeter macrocephalus in IUCN Red List of Threatened Species 8235. doi: 10.2305/IUCN.UK.2008.RLTS.T41755A160983555.en

[ref116] R Core Team , (2023). *R: A language and environment for statistical computing*. R Foundation for Statistical Computing https://www.r-project.org/

[ref117] Teloni V, Mark JP, Patrick MJO, Peter MT (2008) Shallow food for deep divers: dynamic foraging behavior of male sperm whales in a high latitude habitat. J Exp Mar Biol Ecol 354: 119–131. 10.1016/j.jembe.2007.10.010.

[ref118] Tyack PL, Johnson M, Soto NA, Sturlese A, Madsen PT (2006) Extreme diving of beaked whales. J Exp Biol 209: 4238–4253. 10.1242/jeb.02505. 17050839

[ref119] Van Der Hoop JM, Fahlman A, Hurst T, Rocho-Levine J, Shorter KA, Petrov V, Moore MJ (2014) Bottlenose dolphins modify behavior to reduce metabolic effect of tag attachment. J Exp Biol 217: 4229–4236. 10.1242/jeb.108225. 25324344

[ref120] Videsen SKA, Simon M, Christiansen F, Friedlaender A, Goldbogen J, Malte H, Segre P, Wang T, Johnson M, Madsen P (2023) Cheap gulp foraging of a giga-predator enables efficient exploitation of sparse prey. Sci Adv 9: eade3889. 10.1126/sciadv.ade3889. 37352356 PMC10289661

[ref121] Villegas-Amtmann S, Schwarz LK, Sumich JL, Costa DP, Peters DPC (2015) A bioenergetics model to evaluate demographic consequences of disturbance in marine mammals applied to gray whales. Ecosphere 6: 1–19. 10.1890/ES15-00146.1.

[ref122] Wahrenbrock EA, Maruschak GF, Elsner R, Kenney DW (1974) Respiration and metabolism in two baleen whale calves. Mar Fish Rev 36: 3–8.

[ref123] Watwood SL, Miller PJO, Johnson M, Madsen PT, Tyack PL (2006) Deep-diving foraging behaviour of sperm whales (*Physeter macrocephalus*). J Anim Ecol 75: 814–825. 10.1111/j.1365-2656.2006.01101.x. 16689963

[ref124] Whitehead H (1996) Babysitting, dive synchrony, and indications of alloparental care in sperm whales. Behav Ecol Sociobiol 38: 237–244. 10.1007/s002650050238.

[ref125] Whitehead H (2003) Sperm Whales: Social Evolution in the Ocean. Chicago University Press, Chicago, IL, USA.

[ref126] Whitehead H (2018). Sperm whale: Physeter macrocephalus. Encyclopedia of Marine Mammals, 919–925.

[ref127] Whitehead H, Shin M (2022) Current global population size, post-whaling trend and historical trajectory of sperm whales. Sci Rep 12: 19468–19412. 10.1038/s41598-022-24107-7. 36376385 PMC9663694

[ref128] Wickham H (2016). ggplot2: Elegant Graphics for Data Analysis. Springer, New York. https://ggplot2.tidyverse.org, 10.1007/978-3-319-24277-4

[ref129] Wickham H, François R, Henry L, Müller K, Vaughan D (2023) *Dplyr: a grammar of data manipulation* (R package version 1.1.4). https://cran.r-project.org/package=dplyr.

[ref130] Williams R, Noren DP (2009) Swimming speed, respiration rate, and estimated cost of transport in adult killer whales. Mar Mamm Sci 25: 327–350. 10.1111/j.1748-7692.2008.00255.x.

[ref131] Williams TM (1989) Swimming by sea otters: adaptations for low energetic cost locomotion. J Comp Physiol A 164: 815–824. 10.1007/BF00616753. 2724187

[ref132] Williams TM, Haun J, Davis RW, Fuiman LA, Kohin S (2001) A killer appetite: metabolic consequences of carnivory in marine mammals. Comp Biochem Physiol A Mol Integr Physiol 129: 785–796. 10.1016/S1095-6433(01)00347-6. 11440865

[ref133] Williams TM, Kendall TL, Richter BP, Ribeiro-French CR, John JS, Odell KL, Losch BA, Feuerbach DA, Stamper MA (2017) Swimming and diving energetics in dolphins: a stroke-by-stroke analysis for predicting the cost of flight responses in wild odontocetes. J Exp Biol 220: 1135–1145. 10.1242/jeb.154245. 28298467

[ref134] Williams TM, Peter-Heide Jørgensen M, Pagano AM, Bryce CM (2020) Hunters versus hunted: new perspectives on the energetic costs of survival at the top of the food chain. Funct Ecol 34: 2015–2029. 10.1111/1365-2435.13649.

[ref135] Wilson RP, Börger L, Holton MD, Scantlebury DM, Gómez-Laich A, Quintana F, Rosell F, Graf PM, Williams H, Gunner R et al. (2020) Estimates for energy expenditure in free-living animals using acceleration proxies: a reappraisal. J Anim Ecol 89: 161–172. 10.1111/1365-2656.13040. 31173339 PMC7030956

[ref136] Wilson RP, White CR, Quintana F, Halsey LG, Liebsch N, Martin GR, Butler PJ (2006) Moving towards acceleration for estimates of activity-specific metabolic rate in free-living animals: the case of the cormorant. J Anim Ecol 75: 1081–1090. 10.1111/j.1365-2656.2006.01127.x. 16922843

[ref137] Wisniewska DMM, Johnson M, Teilmann J, Rojano-Doñate L, Shearer J, Sveegaard S, Miller LAA, Siebert U, Madsen PTT (2016) Ultra-high foraging rates of harbor porpoises make them vulnerable to anthropogenic disturbance. Curr Biol 26: 1441–1446. 10.1016/j.cub.2016.03.069. 27238281

[ref138] Yazdi P, Kilian A, Culik BM (1999) Energy expenditure of swimming bottlenose dolphins (Tursiops truncatus). Mar Biol 134: 601–607. 10.1007/s002270050575.

