# Supplementary material for: Estimating energy expenditure of sperm whales living in social units
Source: Conserv Physiol. 2026 Apr 29;14(1):coag024. doi: 10.1093/conphys/coag024 (PMC13128299; doi:10.1093/conphys/coag024)
Supplement: Web_Material_coag024 [file web_material_coag024.zip › Supp_Material_MPS_etal_Revision_Clean.pdf]

The following supplementary materials accompanies the article

Estimating energy expenditure of social units of sperm whales

Mariana P. Silva[1*], Cláudia Oliveira[1], Rui Prieto[1], Austin Allen[2], Matthew Bowers[3], Aimee-Kate Darias-O'Hara[4], Andreas Fahlman[5,6,7], Katarína Klementisová[4], Madalina Matei[4], Samantha E. Simmons[4], Mónica A. Silva[1], Leslie New[8], Sergi Pérez-Jorge[1]

Supplementary Materials

Data

We have a total of 84 hours of DTAG data for 7 individuals for the years 2018 and 2019.

Table S1. Data from DTAGs deployed on sperm whales (n=7) in the Azores archipelago (years 2018-2019) including duration (in hours) of tag deployment, number of foraging dives, proportion of time (%) spent diving, on descent, on bottom, on ascent and on surface periods 1, 2 and 3. Surface periods 1 and 2, correspond to the initial 80% and the final 20% of surface time following a dive (up to 9 minutes), respectively. Surface periods longer than 9 minutes were composed of three parts: surface periods 1 and 2 (the initial 9 minutes, maintaining the same physiological distinction as in shorter post-dive periods) and, surface period 3 corresponding to a non-foraging surface period (any remaining time beyond the 9-minute threshold, not directly associated with immediate post-dive recovery but rather with other behaviours such as resting, socializing or traveling). This table also includes estimates of OBDA (g) for each individual.

Individual ID	Tag duration (h)	Foraging dives (n)	Proportion of time spent in each activity in relation to tag duration (%)						ODBA	
			dives	descent	bottom	ascent	Surface periods 1 and 2	Surface period 3	Mean	SD
2	4.46	1	19.80	5.15	11.24	3.41	0.00	80.20	0.13	0.21
3	5.15	3	52.06	11.09	19.94	7.35	13.74	47.93	0.09	0.37
4	22.04	22	90.67	15.31	47.52	13.84	14.10	9.33	0.09	0.17
5	8.27	8	77.12	13.63	36.67	14.19	12.38	23.24	0.09	0.15
6	6.67	6	88.55	11.79	50.54	11.28	14.77	11.67	0.06	0.11
7	24.69	22	85.78	12.94	49.77	10.37	12.71	14.33	0.07	0.07
8	12.02	10	81.51	13.49	43.13	10.17	14.84	18.48	0.07	0.08

Length-weight distributions

We used an average body length (L_{body}) of 861 (±89) cm (estimated from photogrammetry measurements of eight individuals in the area - unpublished data) to create a distribution of length values for social units of sperm whales in the Azores (Table S2, Fig. S1A). These values were used to simulate a distribution of body mass (M_{body}) values (Table S2 Fig. S1B) with a length-mass relationship (Lockyer, 1976), as follows:

$$M_{body} = 1.25 \times 0.0196 \, L_{body}^{2.74} \qquad (S1)$$

The resulting M_{body} distribution was right-skewed due to the nonlinear exponent, so the 95% confidence interval was calculated directly from the 2.5th and 97.5th percentiles of the simulated values (Table S2).

Table S2. Length and weight estimated in this study for sperm whales from social units in the Azores.

Parameter	Value	Notes
Length, L_{body}	861 (±89)<br>95% CI: 6.86-10.36	Mean (±SD) from photogrammetry.<br>95% CI estimated from simulated distribution of length values for Azores
Weight, M_{body}	9167.37<br>95% CI: 4792.574-14832.85	Distribution of weight values for Azores. Derived from simulated L_{body} values using Equation S1. Mean and 95% CI based on simulated mass distribution (non-normal).

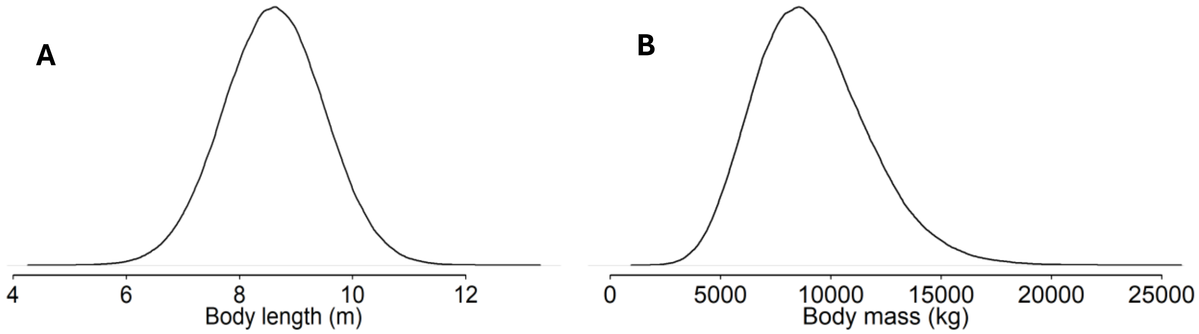

Figure S1. A) Distribution of body length (L_{body}) using a mean of 8.61 (±0.89) estimated from photogrammetry measurements of eight individuals - unpublished data. **B)** Simulated distribution of body mass (M_{body}) obtained from length-mass relationships (Lockyer, 1976).

Respiration Rates

Respiration rate (f_R) was assessed during surfacing periods following dives in three individuals (Fig. S2. A, B and C). The durations of these three additional deployments ranged from 3.6–19 hours, with one deployment extending into both daytime and nighttime periods. Sperm whales are known to forage throughout the diel cycle, with no consistent differences in diving behaviour or foraging effort between day and night (Aoki et al., 2012; Watwood et al., 2006). Moreover, because respiration rates were analysed separately for foraging and non-foraging phases, any potential diel differences are likely reflected in these behavioural categories, as resting behaviour typically occurs during non-foraging periods.

The analysis showed that f_R (mean=5.06, SD=0.93 was higher during the initial 80% of the surface period before decreasing in the remaining time (mean=3.5, SD=1.14). Based on this pattern, these surface periods were divided into two phases: surface period 1 and 2, defined as the initial 80% and the final 20% of surface time following a dive, respectively. Notably, f_R in surface period 1 was significantly higher than in surface period 2 (Wilcoxon test, $W=7353.5$, $p<0.05$).

Table S3. Data from individuals which the DTAGs were deployed near the blowhole (n=3), allowing for a more accurate respiration detection.

Individual ID	Tag duration (h)	Day time (h)	Night time (h)	Foraging dives (n)	Post-dive surface periods (n)
sw22_234a	03:35:15	03:35:15	none	3	3
pm10_222a	05:16:58	05:16:58	none	7	7
pm10_228a	18:57:05	09:19:49	09:37:16	20	20

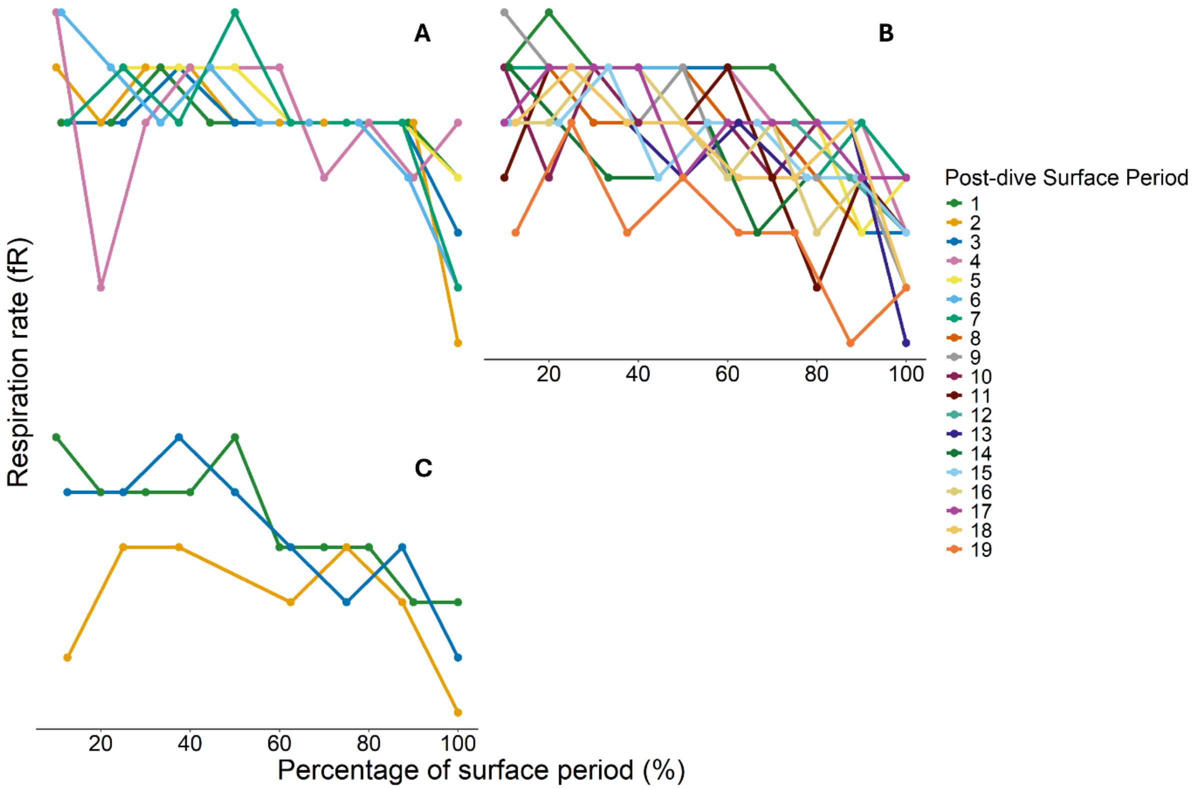

Figure S2. Respiration rate (f_R) from post-dive surface periods from three individuals: A (pm10_222a), B (pm10228a) and C (sw22_234a) with the DTAGs positioned near the blowhole.

Sensitivity analysis

We conducted a sensitivity analysis to explore the uncertainty associated with V_t, and E_{O2} when estimating FMR. In this analysis, each parameter (either V_t or E_{O_2}) was systematically varied across a biologically relevant range across the three defined surface periods (1, 2, and 3), while the other was held constant at its default model value (Table S4). The remaining surface periods retained their original parameter values (Table 1) to assess the independent effect of each variation. This approach resulted in six sensitivity scenarios covering variations in both V_t and E_{O_2} (Table S4). For each scenario, 1000 Monte Carlo simulations were performed to evaluate the impact of parameter changes on FMR estimates. The modified parameters are highlighted in Table S4, while unmodified parameters remain consistent with the baseline model values.

Table S4. Sensitivity of FMR estimates. For each scenario 1000 simulations were run. The modified parameter is highlighted in grey/bold.

Scenario	Surface period where the change happens	V_t (% V_c)	E_{O2} (%)	FMR			
				mean	SD	CI lower	CI upper
1	1	**40-80**	80	365.40	0.09	204.19	581.26
2	1	70	**50-90**	371.27	0.08	239.53	552.47
3	2	**20-60**	50	409.85	0.09	259.44	613.51
4	2	45	**20-60**	407.83	0.09	258.13	611.01
5	3	**10-50**	35	403.73	0.09	251.34	610.09
6	3	35	**10-50**	403.73	0.09	256.14	604.01

Current model FMR estimates = 412.43 MJ/day (95% CI: 263.17-614.12) e.g., using values from Table 1.

Overall dynamic body acceleration (ODBA)

To account for variability in the different dive phases when simulating random dives, we created distributions of duration and ODBA for each dive phase (descent, bottom, ascent) (Fig. S3). The mean and standard deviation of the distributions were informed by the data on durations of each phase and ODBA obtained from tagged whales in this study. The correlation included between dive durations across the different dive phases was estimated based upon the observed correlations within the data.

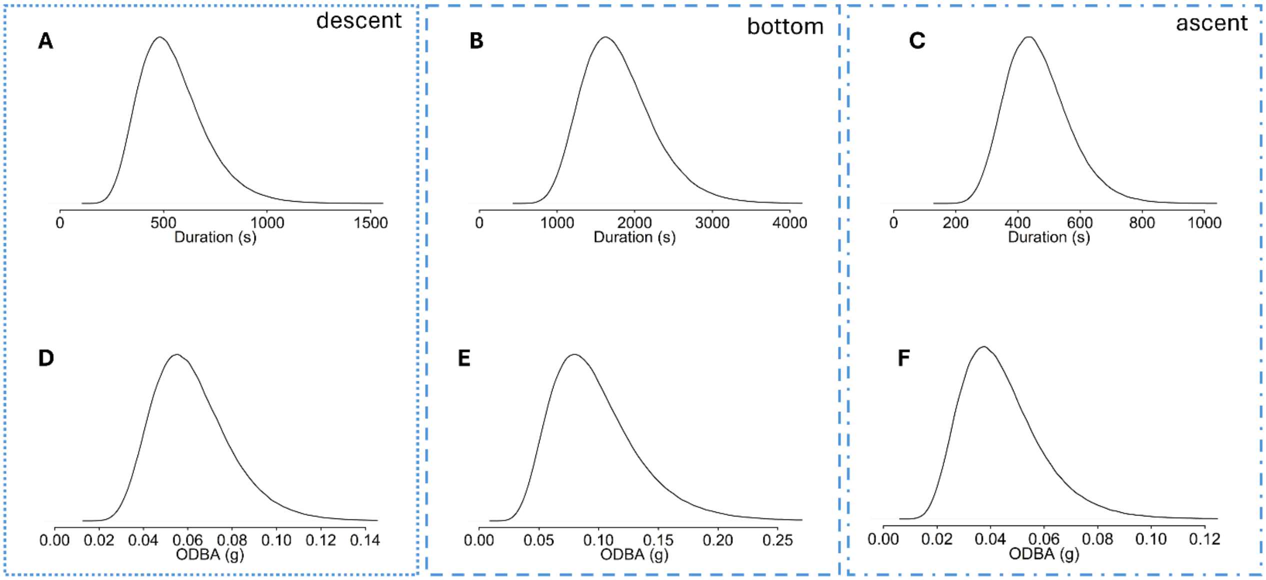

Figure S3. Distribution of parameters for each dive phase: descent (dotted outline), bottom (dashed outline) and ascent (dash dot outline). **A, B** and **C)** mean duration (in sec). **D, E** and **F)** mean ODBA (in g).

Mean field metabolic rate (FMR)

Table S5. Mean field metabolic rate (FMR, MJ/day) of cetaceans and their corresponding multiples of the basal metabolic rate (BMR) predicted by Kleiber's equation for terrestrial mammals (Kleiber, 1975). For species with more than one published estimate, FMR values represent the mean across references. FMR values for sperm whales (this study) and baleen whales were obtained from modelling approaches. The beluga FMR represents a theoretical estimate (3xRMR) from captive animals. Harbour porpoise data include both free-ranging and captive individuals using doubly labelled water (DLW) and respiration rate methods. Bottlenose dolphin FMR was measured in captive animals using DLW method. Orcas' FMRs were modelled from activity-specific swimming speeds and the cost of transport regressions from (Williams & Noren, 2009).

Species	Mbody (kg)	FMR (MJ/day)	BMR (MJ/day)	Mean FMR as multiple of Kleiber	References
Sperm whale	9163	412.27	272.52	1.59	Our study – using respiration rates
Sperm whale	9163	620.5	272.52	2.39	Our study – using ODBA
Orca	3886.00	906.88	143.99	6.17	(Noren, 2011)
Beluga	758.00	215.47	42.34	5.09	(John, 2020; John et al., 2024)
Bottlenose	214.52	53.36	16.43	3.25	(Rimbach et al., 2021)
Harbour porpoise	60	21.7	6.32	3.43	(Rojano-Donãte et al., 2018)
Humpback whale	21682.30	1629.18	523.71	3.11	(Blawas et al., 2025)
Minke whale	4469.70	353.03	160.06	2.20	(Blawas et al., 2025; Blix & Folkow, 1995)
Blue whale	71647.14	3505.54	1283.56	2.73	(Blawas et al., 2025)
Southern right whale	31886.00	687.50	699.38	0.98	(Christiansen et al., 2023)

Table S6. Previously reported relationships between field metabolic rate (FMR) and body mass (M_{body}) for marine mammals and terrestrial carnivores.

MJ/day	Context	References
$FMR = 0.5972863 \times M_{body}{}^{0.808}$	All mammals	Nagy (2005)
$FMR = 1.69776 \times M_{body}{}^{0.756}$	killer whales	Williams et al. (2004)
$FMR = 3.3.062688 \times M_{body}{}^{0.49}$	For marine mammals	Maresh 2014
$FMR = 0.7451704 \times M_{body}{}^{0.49}$	For terrestrial carnivores	Maresh 2014
$FMR = 0.53 \times M_{body}{}^{0.90}$	porpoises	Rojano-Donate et al. 2018
$FMR = 0.6845 \times M_{body}{}^{0.80}$	For marine mammals	William et al. 2020
$FMR = 0.6512 \times M_{body}{}^{0.87}$	For terrestrial carnivores	William et al. 2020

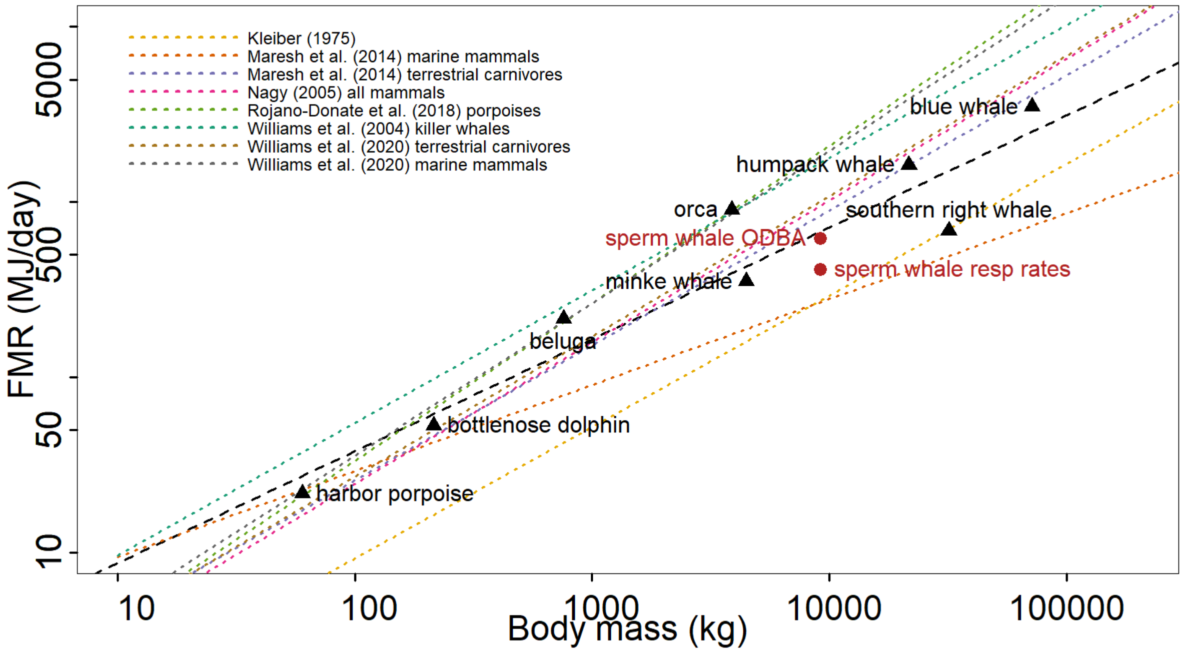

Figure S4. Logarithmic relationship between body mass (M_{body}) and field metabolic rate (FMR) for sperm whales (red circles) and other cetaceans (black triangles). Each point represents a species-specific FMR value calculated as the mean when multiple estimates were available. The estimate for the beluga whale represents a theoretical estimate (3xRMR) due to the absence of daily FMR data for this species. Dashed black solid line is the regression through the cetacean data points. The yellow dotted line is the Kleiber (1975) allometric regression of basal metabolic rate in relation to body mass for terrestrial mammals. Other coloured dashed lines: previously proposed relationships of FMR and M_{body} for marine mammals and terrestrial carnivores. Data and original reference sources are provided in tables S5 and S6.

References

Aoki, K., Amano, M., Mori, K., Kourogi, A., Kubodera, T., & Miyazaki, N. (2012). Active hunting by deep-diving sperm whales: 3D dive profiles and maneuvers during bursts of speed. *Marine Ecology Progress Series*, *444*, 289–301. https://doi.org/10.3354/meps09371

Blawas, A. M., Videsen, S. K. A., Cade, D. E., Calambokidis, J., Friedlaender, A. S., Johnston, D. W., Madsen, P. T., & Goldbogen, J. A. (2025). Life in the slowest lane: Feeding allometry lowers metabolic rate scaling in the largest whales. *Science Advances* , *11*(32). https://doi.org/10.1126/sciadv.adw2232

Blix, A. S. S., & Folkow, L. P. (1995). Daily energy expenditure in free living minke whales. *Acta Physiologica Scandinavica*, *153*(1), 61–66. https://doi.org/10.1111/j.1748-1716.1995.tb09834.x

Christiansen, F., Sprogis, K. R., Nielsen, M. L. K., Glarou, M., & Bejder, L. (2023). Energy expenditure of southern right whales varies with body size, reproductive state and activity level. *Journal of Experimental Biology*. https://doi.org/10.1242/jeb.245137

John, J. S. (2020). *Energetics of rest and locomotion in diving marine mammals: novel metrics for predicting the vulnerability of threatened cetacean, pinniped, and sirenian species*. University ofCalifornia, Santa Cruz.

John, J. S., Christen, D. R., Flammer, K. L., Kendall, T. L., Nazario, E. C., Richter, B. P., Gill, V., & Williams, T. M. (2024). Conservation energetics of beluga whales: using resting and swimming metabolism to understand threats to an endangered population. *The Journal of Experimental Biology*, *227*(5). https://doi.org/10.1242/jeb.246899

Kleiber, M. (1975). Metabolic turnover rate: A physiological meaning of the metabolic rate per unit body weight. *Journal of Theoretical Biology*, *53*(1), 199–204. https://doi.org/10.1016/0022-5193(75)90110-1

Lockyer, C. (1976). Body weights of some species of large whales. *ICES Journal of Marine Science*, *36*(3), 259–273. https://doi.org/10.1093/icesjms/36.3.259

Noren, D. P. (2011). Estimated field metabolic rates and prey requirements of resident killer whales. *Marine Mammal Science*, *27*(1), 60–77. https://doi.org/10.1111/j.1748-7692.2010.00386.x

Rimbach, R., Amireh, A., Allen, A., Hare, B., Guarino, E., Kaufman, C., Salomons, H., & Pontzer, H. (2021). Total energy expenditure of bottlenose dolphins (Tursiops truncatus) of different ages. *Journal of Experimental Biology*, *224*(15). https://doi.org/10.1242/jeb.242218

Rojano-Donãte, L., McDonald, B. I., Wisniewska, D. M., Johnson, M., Teilmann, J., Wahlberg, M., Højer-Kristensen, J., & Madsen, P. T. (2018). High field metabolic rates of wild harbour porpoises. *Journal of Experimental Biology*, *221*(23), 1–4. https://doi.org/10.1242/jeb.185827

Watwood, S. L., Miller, P. J. O., Johnson, M., Madsen, P. T., & Tyack, P. L. (2006). Deep-diving foraging behaviour of sperm whales (Physeter macrocephalus). *Journal of Animal Ecology*, *75*(3), 814–825. https://doi.org/10.1111/j.1365-2656.2006.01101.x

Williams, R., & Noren, D. P. (2009). Swimming speed, respiration rate, and estimated cost of transport in adult killer whales. *Marine Mammal Science*, *25*(2), 327–350. https://doi.org/10.1111/j.1748-7692.2008.00255.x
